# Supplementary material for: Genetic basis of right and left ventricular heart shape
Source: Nat Commun. 2024 Nov 14;15:9437. doi: 10.1038/s41467-024-53594-7 (PMC11564811; doi:10.1038/s41467-024-53594-7)
Supplement: Supplementary file 1 — Supplementary Information [file 41467_2024_53594_MOESM1_ESM.pdf]

Supplementary

Methods

Quality control of the modelling cohort

Firstly, cases that had an incomplete set of valve landmarks, or incomplete machine learning contours for 3D modelling, were excluded. Four types of shape-based quality scores were computed:

1. Model-contour error in mm, being the average distance between the contour points and the closest model surface points.
2. Individual principal component z-scores for the first 10 components.
3. Mahalanobis distance over the first 10 principal components, being the root sum of squares of the first 10 z-scores.
4. Residual error in mm between the model and its projection onto the first 10 modes.

In each case, scores 5 interquartile ranges above the lower quartile and below the upper quartile were excluded.

Contour studies in the UK Biobank cohort were checked for completeness; the guide point files generated internally post contouring were parsed to count the number of valve points extracted from the contour data at the timeframe marked as end-diastole. As the valve points are plotted on the LAX images, a check for a complete set of 10 valve points (6 mitral comprising 2 from 2-chamber, 2 from 3-chamber, 3 from 4-chamber, 2 aortic from the 3-chamber, 2-tricuspid from the 4-chamber) serves as a check of whether an imaging study for a participant has a complete set of LAX images (which are vital for model construction). We identified 2740 such cases with an incomplete set of LAX images. The other check was performed by parsing the log files generated from the cvi42 software to identify cases missing a significant amount of SAX images and contours. A lack of SAX contours in the modelling process can lead to a severely misshapen or de-personalized end-diastolic model. We identified 317 of these cases.

Model surface-contour distance error

This score was an averaged calculation of the distance between each contour in the end-diastolic study and its closest point on the surface of the fitted model. As the model surface is made up of triangles, calculating the distance between a contour and the surface of the model can give insight into how well the model is fitting the contour points. This is a Euclidean distance calculated in mm for each contour

point and averaged to give an overall metric of the model fit. We identified 836 models in which the total distance score exceeded the interquartile range boundary.

Individual z-scores

These are the raw phenotypes used in the genetic analyses. A Z-score is a measure of a value's relationship to the mean in a distribution; it measures standard deviations from the mean. In the context of the cardiac atlas a Z-score for a principal component describes a model's variation from the mean shape of the atlas in the direction of the shape variation captured by that PC (e.g., in PC1 - the overall size of the heart, a Z-score would relate to whether an individual model was bigger or smaller than the mean shape). Severe outliers in Z-score are indicative of errors in the modelling process rather than biologically significant findings and are important to exclude. In total we identified 279 models with Z-scores that exceeded the interquartile range boundary. These Z-scores are computed through PCA, and those that fail are not included in the GWAS.

Mahalanobis distance

Mahalanobis distance is described as a multivariate equivalent of Euclidean distance. As a metric it is much better suited to multivariate data than measures such as Euclidean distance; it is effective regardless of whether variables are correlated or differently scaled through the use of covariance matrices. The formula for calculating the distance is as follows:

$$D^2 = (x - m)^T . C^{-1} . (x - m)$$

Where:

- D^2 is the square of the Mahalanobis distance
- x is the vector of the observation (row in a dataset)
- m is the vector of mean values of independent variables (mean of each column)
- C^{-1} is the inverse covariance matrix of independent variables

In our cohort we identified 831 cases as multivariate outliers exceeding the five interquartile range boundaries in their Mahalanobis distance when applied to the 10 PC scores.

Residual error in component space

We also computed the error between each individual shape and its projection onto the first 10 modes, to provide an estimate of residual distance not included in the above metrics. We identified 605 cases exceeding the 5IQR threshold.

Selection of analysis phenotypes

The first 11 PCs were selected as phenotypes for the genomics analyses. It is difficult to determine how many PCs to include since each subsequent principal component captures less shape variation observed in the atlas. The components past PC11 capture < 1% of shape variation observed in the atlas. Also, the levels of estimated heritability (the proportion of variation in the phenotype that can be attributed to genetic variation rather than environmental variation) decrease with further PCs.

Major adverse cardiovascular event (MACE) definition

The study cohort used in the genome-wide association study selected healthy European UK Biobank participants with no recorded major adverse cardiovascular events. This was defined using hospital episode statistics data ICD10 codes: I20.0, I21, I22, I23, I24, I26, I30, I40, I41, I33, I38, I43, I46, I51, I44, I45, I47, I48, I49, I50, I60, I61, I62, I63, I64, I69, I710, I711, I713, I715, I718, I42, I13, I14. We also use ICD9 codes 4109, 4280, 4281, 4289. Finally, we also exclude participants with self-reported previous major adverse cardiovascular events from the baseline questionnaire, as well as participants with either self-reported or algorithmically defined myocardial infarction or heart failure using the relevant UK Biobank data fields.

Shape PC exploration

Here we describe the author’s interpretation of the first 11 PCs in the shape atlas, the shape variation observed in each PC and the effect of a positive/negative Z-score on the shape. GIF representations of these PCs are available in the supplementary to better visualize the shape variations alongside these descriptions.

1. Overall heart size. A bigger Z score corresponds to a bigger heart, a smaller Z score corresponds to a smaller heart.
2. Apex to base length. We observe significant variation in the aorta position and apical position leading to variation in heart height. A bigger Z score reflects lower apex and longer ventricle, with a smaller Z score reflecting shorter, wider ventricles.
3. Width in the anterior-posterior direction. With a higher Z score we observe a wider heart in the anterior-posterior direction, with a lower Z score we observe a thinner heart in the anterior-posterior dimension.
4. Orientation of the right ventricle relative to left. With a higher Z score we observe a lower tricuspid valve, more normal tricuspid cut (perpendicular to the apex-base axis), with a lower Z

score we see the valves closer together, squaring off in the base and the tricuspid valve sitting higher.

5. Height to width ratio in the lateral dimension. With a lower Z score we see a shorter, wider heart, and with a higher Z score we see the ventricles thinner and longer.
6. Right ventricular torsion of the tricuspid valve. With a higher Z score we see the tricuspid valve wrapped towards the left ventricle, whereas with a lower Z score we see the tricuspid valve oriented further away from the left ventricle.
7. Right ventricular inferior margin bulging. With a higher Z score we see a higher mitral valve position, and with a lower Z score we see a lower apex alongside a raised tricuspid valve and a bulge in the right inferior margin.
8. Left ventricular morphology. With a higher Z score we observe a wider tricuspid valve, thinner left ventricular morphology, less raised mitral, lower Z score smaller tricuspid opening, wider left ventricular morphology and raised mitral valve.
9. LV size and roundness. A lower Z score corresponds with a lower apical position and longer left ventricle, whereas a higher Z score corresponds to a higher apical position and shorter left ventricular morphology.
10. Left ventricular lateral apex displacement. With a higher Z score we observe a higher mitral valve leaflet at edge, thicker LV wall and a lower left ventricular apex wrapping around the apex of the right ventricle.
11. Left ventricular free wall thickness. Positive Z scores were associated with left ventricular free wall thinning and septal bulge into the right ventricle alongside some RV base rounding. Lower Z scores were associated with a thicker left ventricular free wall and wider valve openings.

Supplementary Figures

Supplementary Figure 1. Genetic correlation matrix between PCs

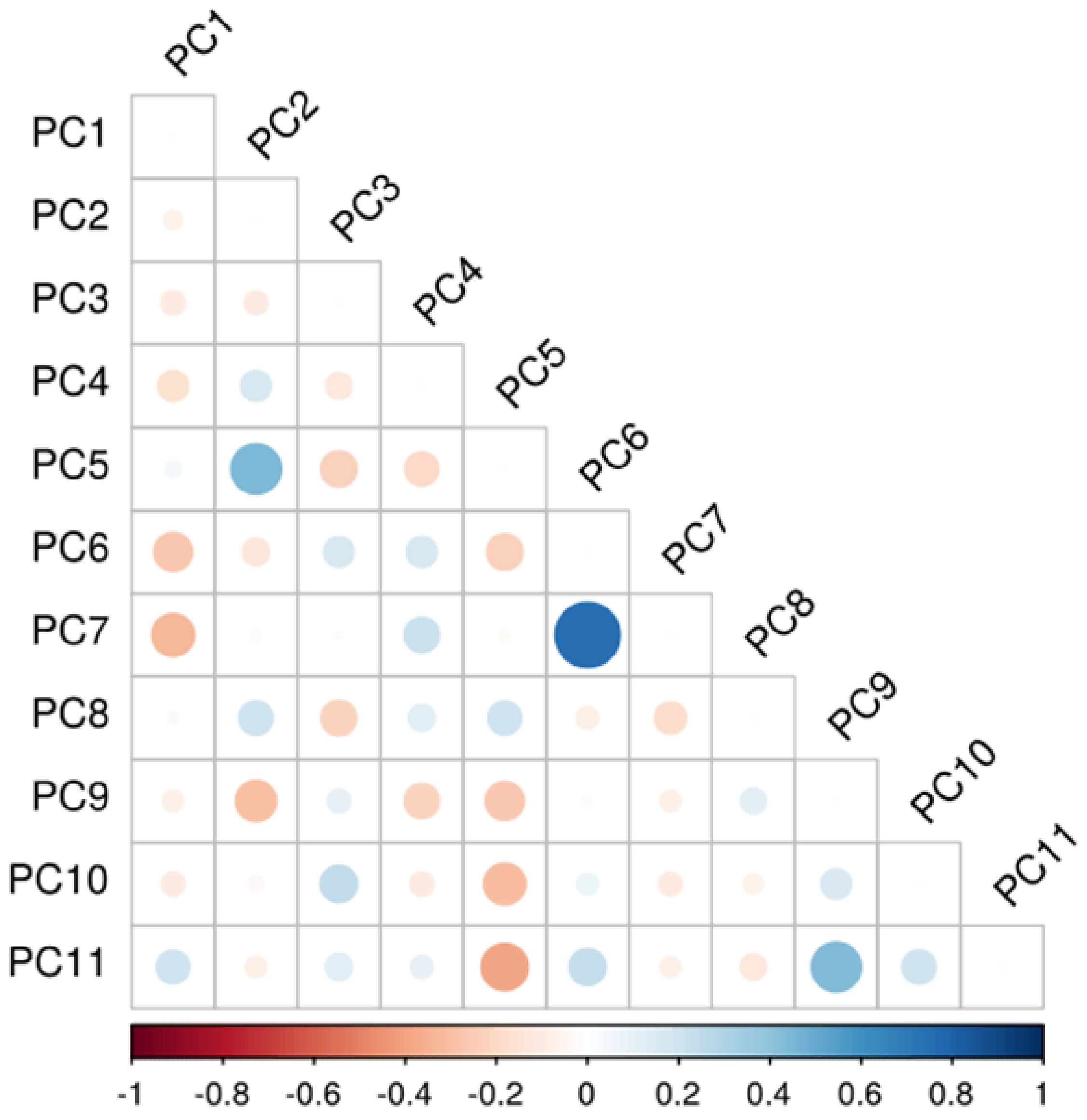

PC, principal component; Corr, correlation; In this correlation matrix the size of the circle indicates the level of p-value significance (a larger circle is a lower p-value), and the color of the circle indicates the strength of the correlation coefficient (red: positive, blue: negative).

Supplementary Figure 2. Quantile-Quantile plots

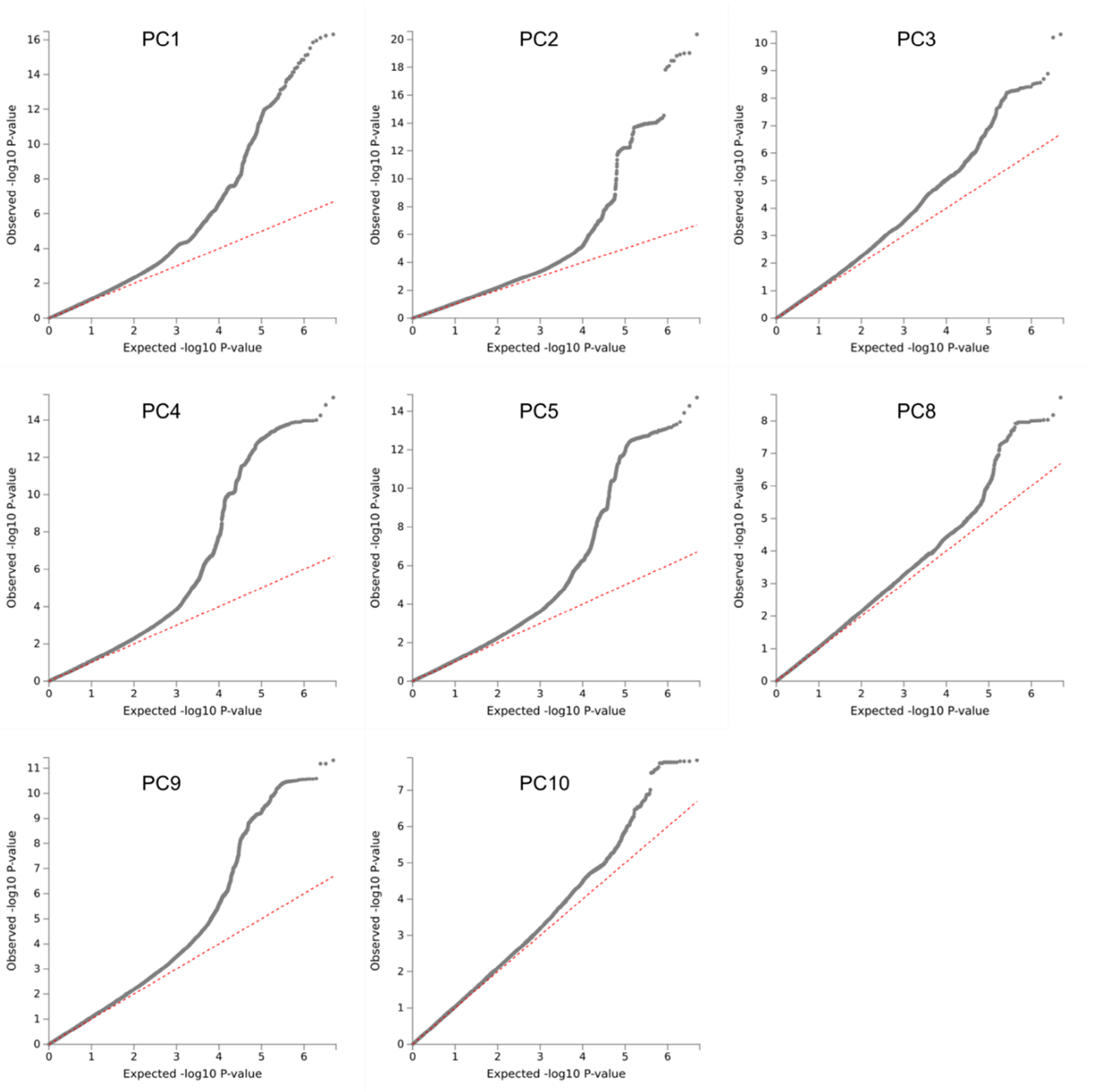

PC, principal component

Supplementary Figure 3. Manhattan plots for PC GWAS

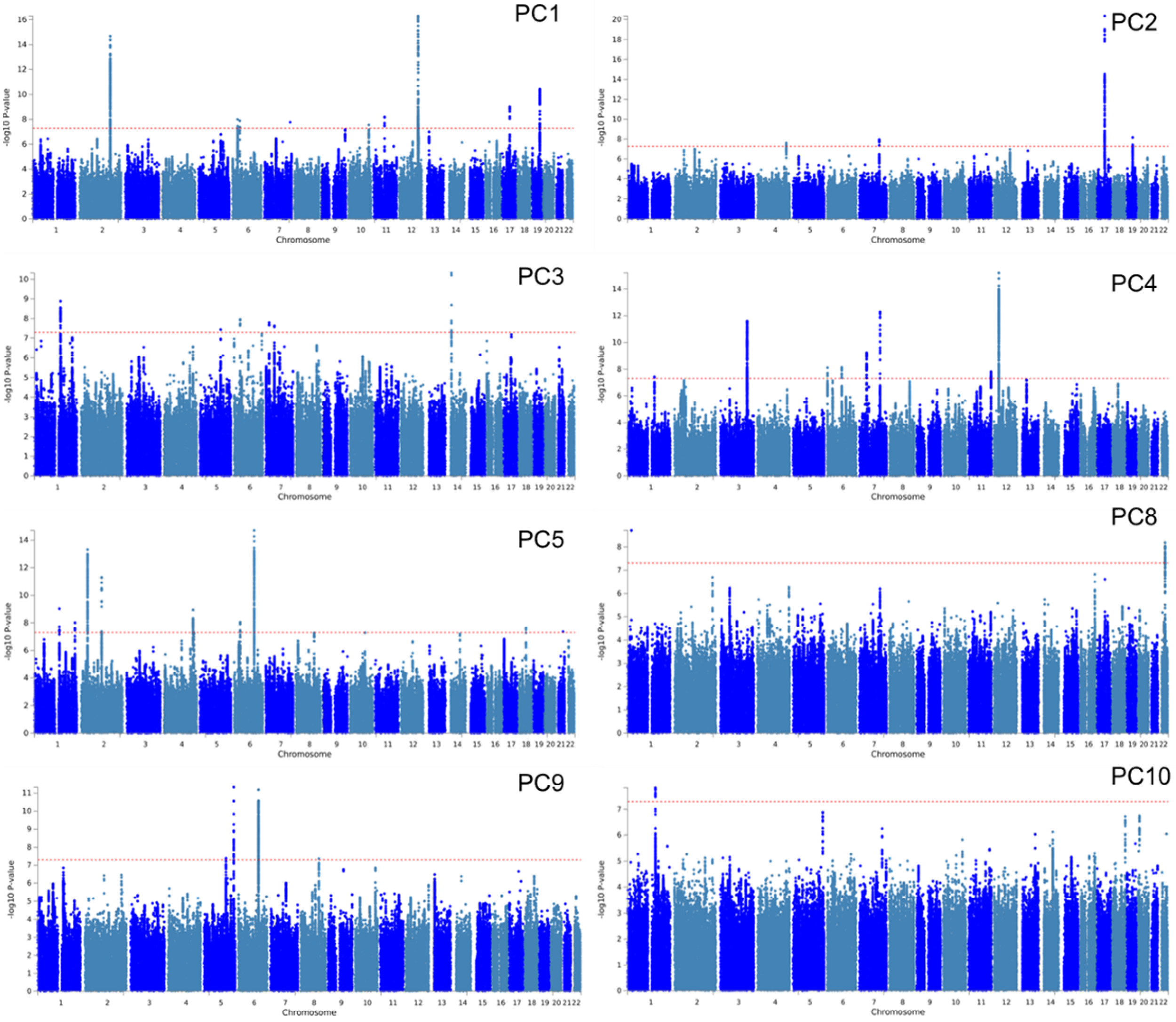

The red dotted line indicates the threshold for genome wide significance (p < 5E-8)

Supplementary Figure 4. GARFIELD GWAS SNV tissue enrichment

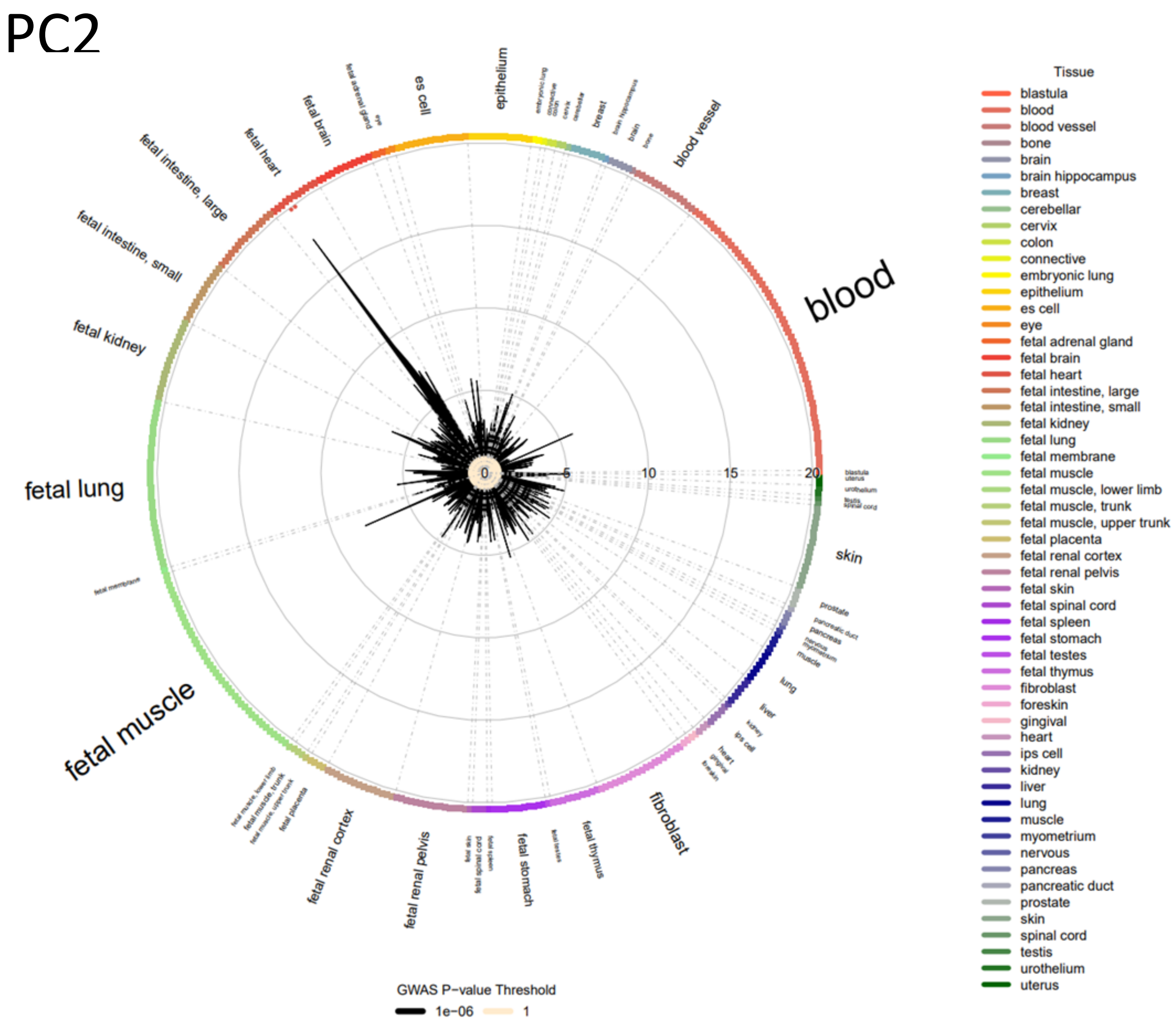

PC5

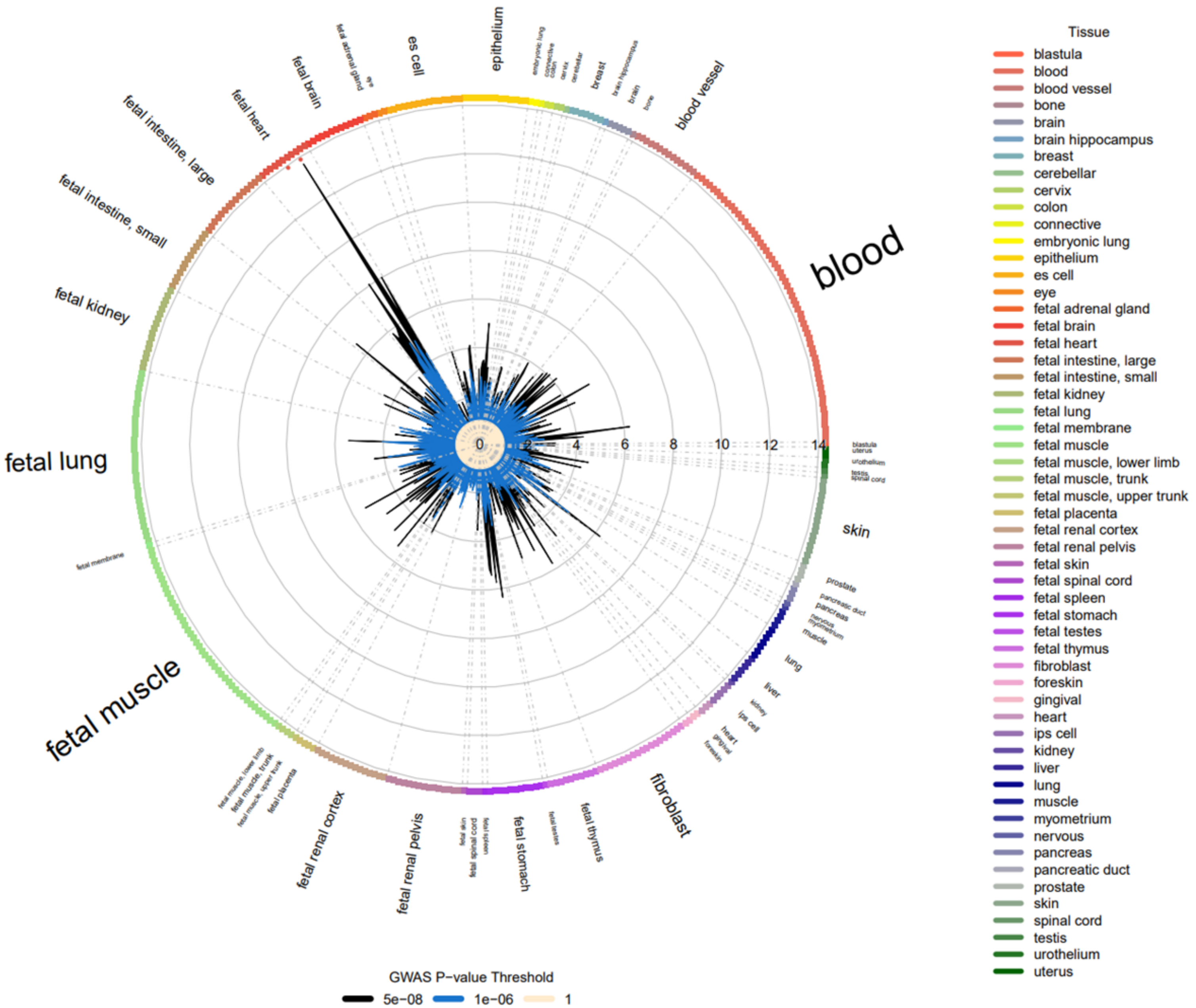

Only PCs 2 & 5 had significant GARFIELD results to report; GWAS, genome-wide association study; GARFIELD, GWAS Analysis of Regulatory and Functional Information Enrichment with LD correction; PC, principal component; tissue types are grouped and colour coded around the circumference, with SNV enrichment illustrated by peaks from the centre, with the height of the peak corresponding to level of significance. Black peaks indicate results from SNV's with GWAS p-value < $1E^{-6}$, and blue indicates SNV's with p-value < $5E^{-8}$. Tissue font size is proportional to the number of cell types for that tissue. Small dots on the inner side of the outer circle show if the observed enrichment is significant in direction outside to inside.

Supplementary Figure 5.1 LocusZoom plot for 10:121347839_TTTTC_T from GWAS for PC1

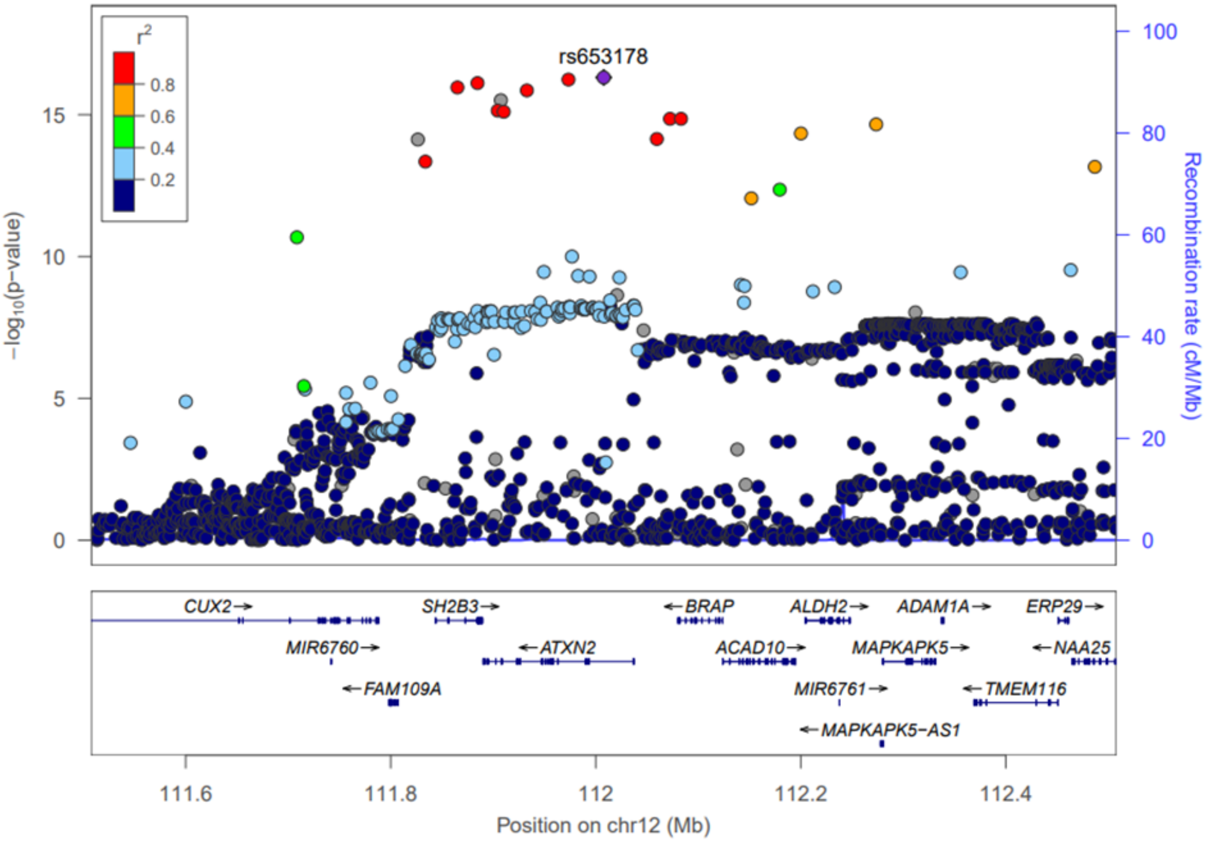

Supplementary Figure 5.2 LocusZoom plot for rs653178 from GWAS for PC1

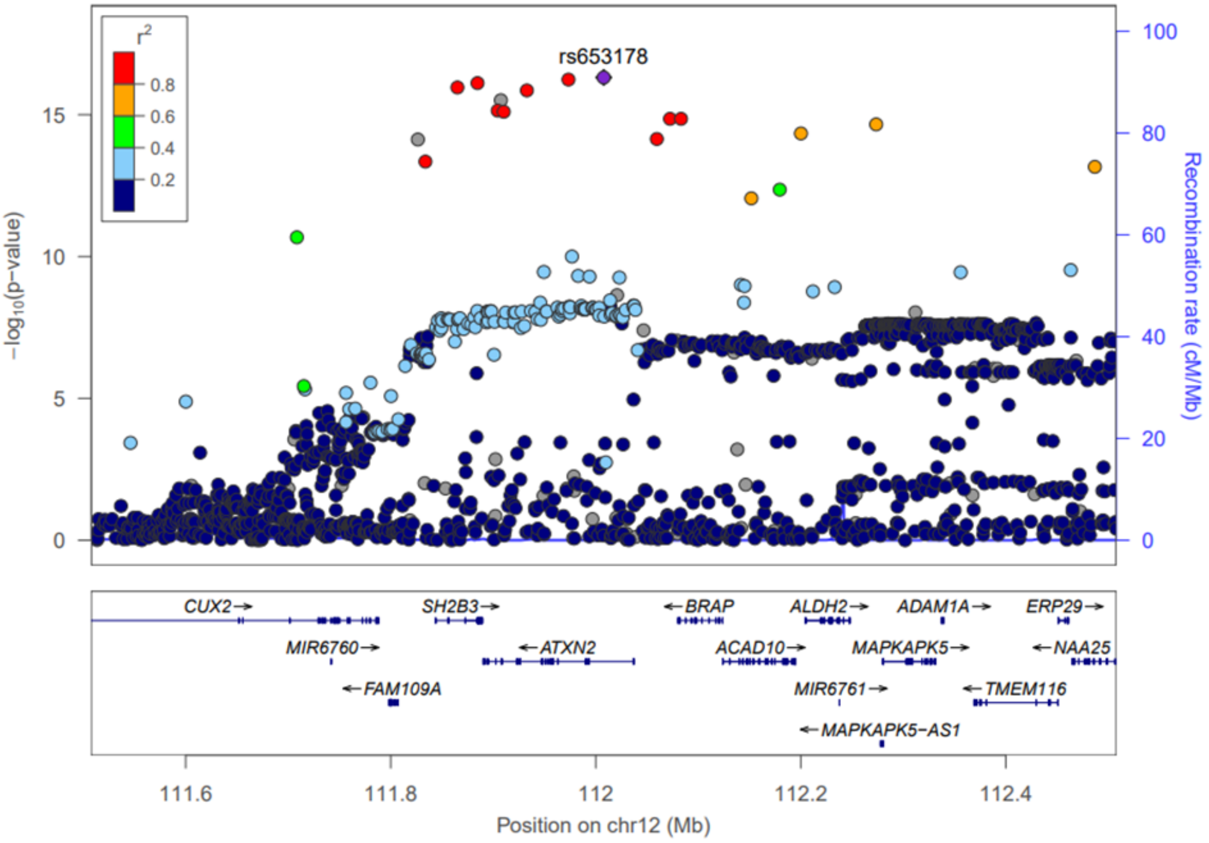

147 **Supplementary Figure 5.3 LocusZoom plot for rs2146324 from GWAS for PC1**

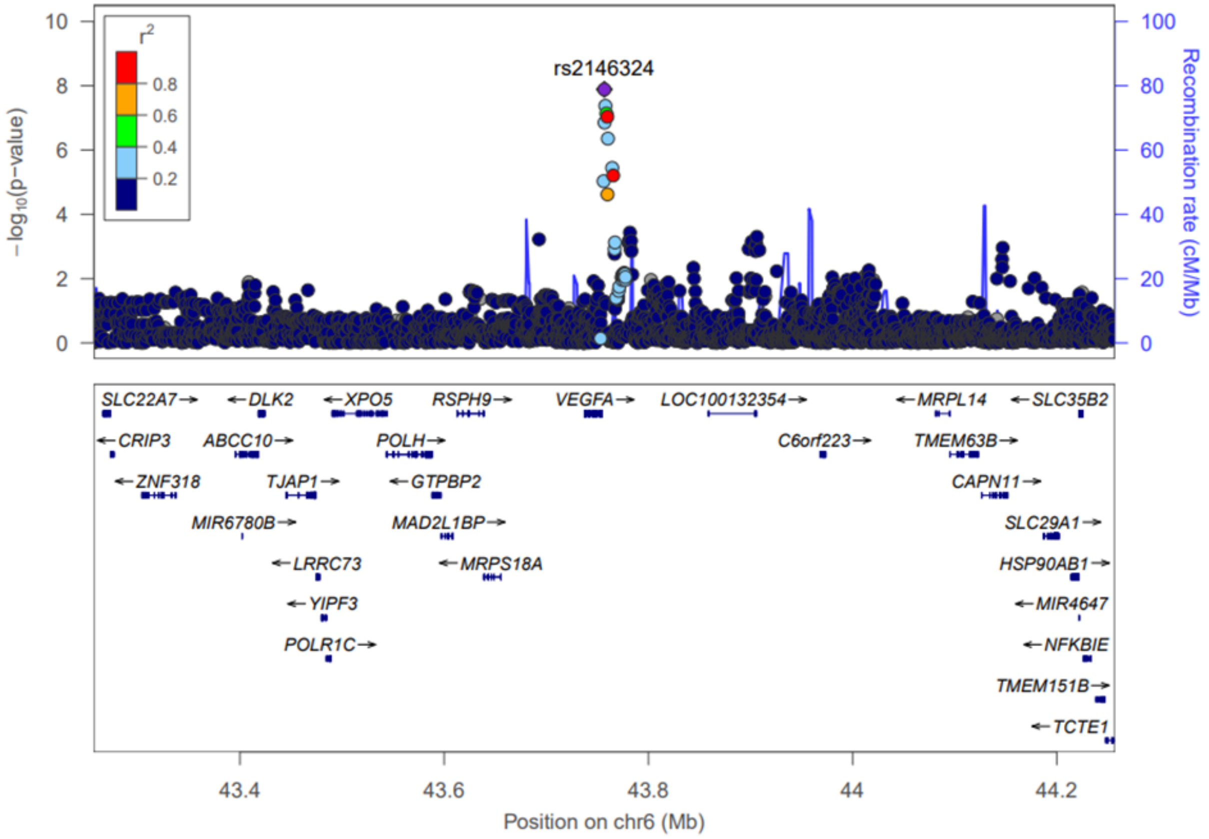

148

149

Supplementary Figure 5.4 LocusZoom plot for rs2279472 from GWAS for PC1

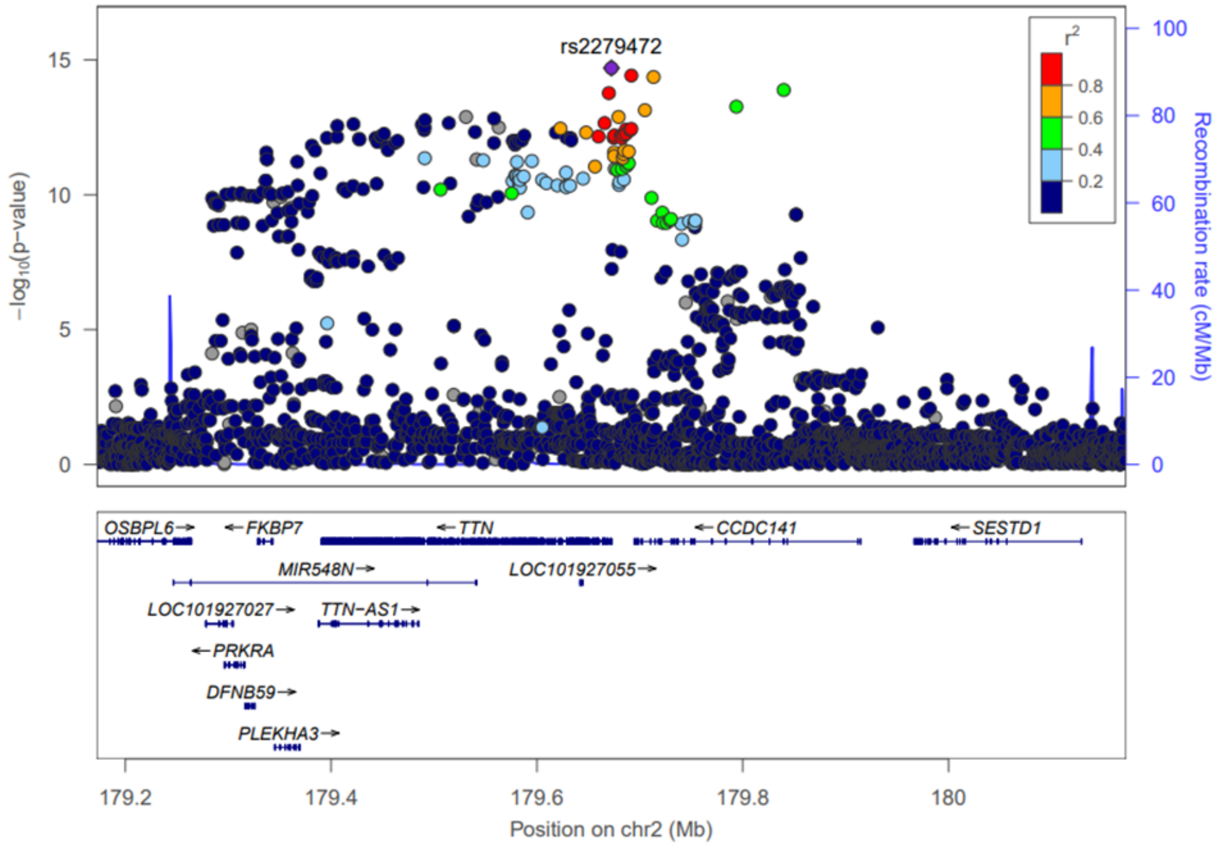

Supplementary Figure 5.5 LocusZoom plot for rs3918226 from GWAS for PC1

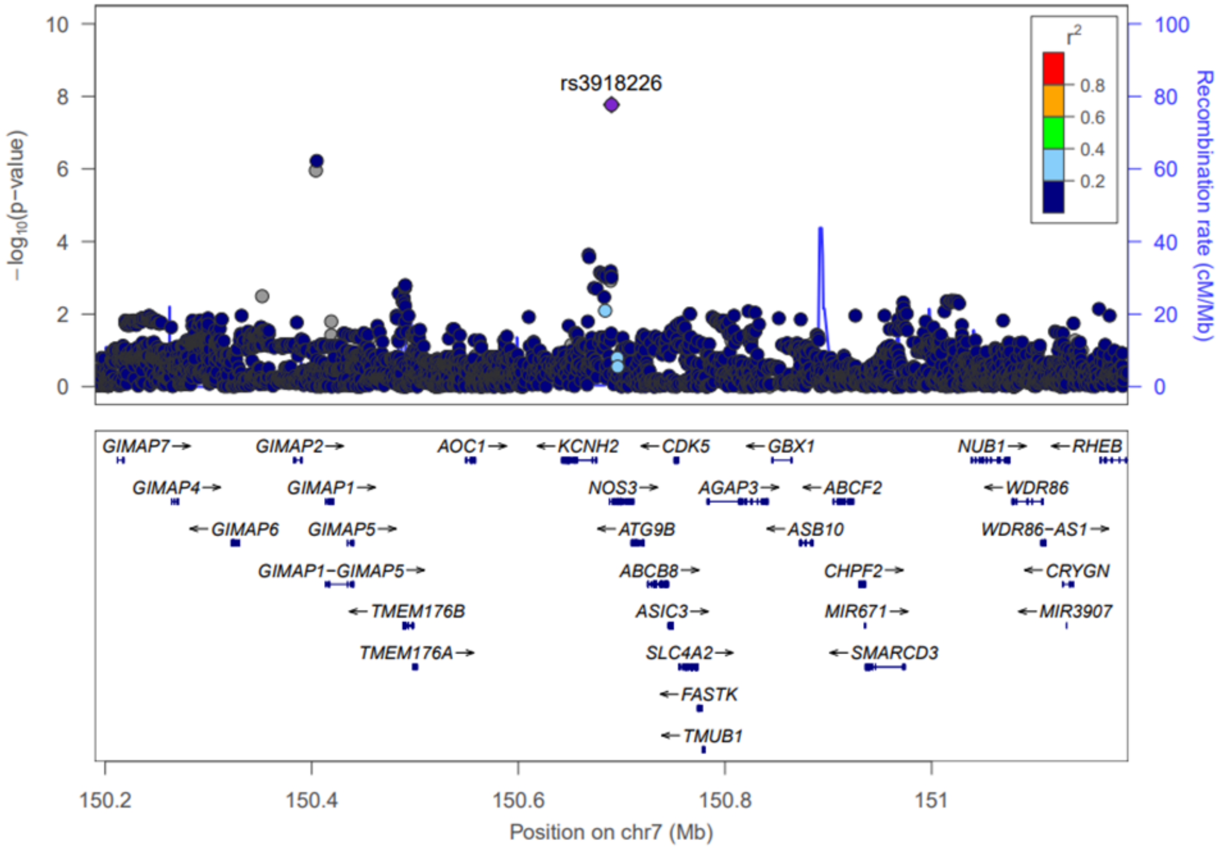

Supplementary Figure 5.6 LocusZoom plot for rs7773935 from GWAS for PC1

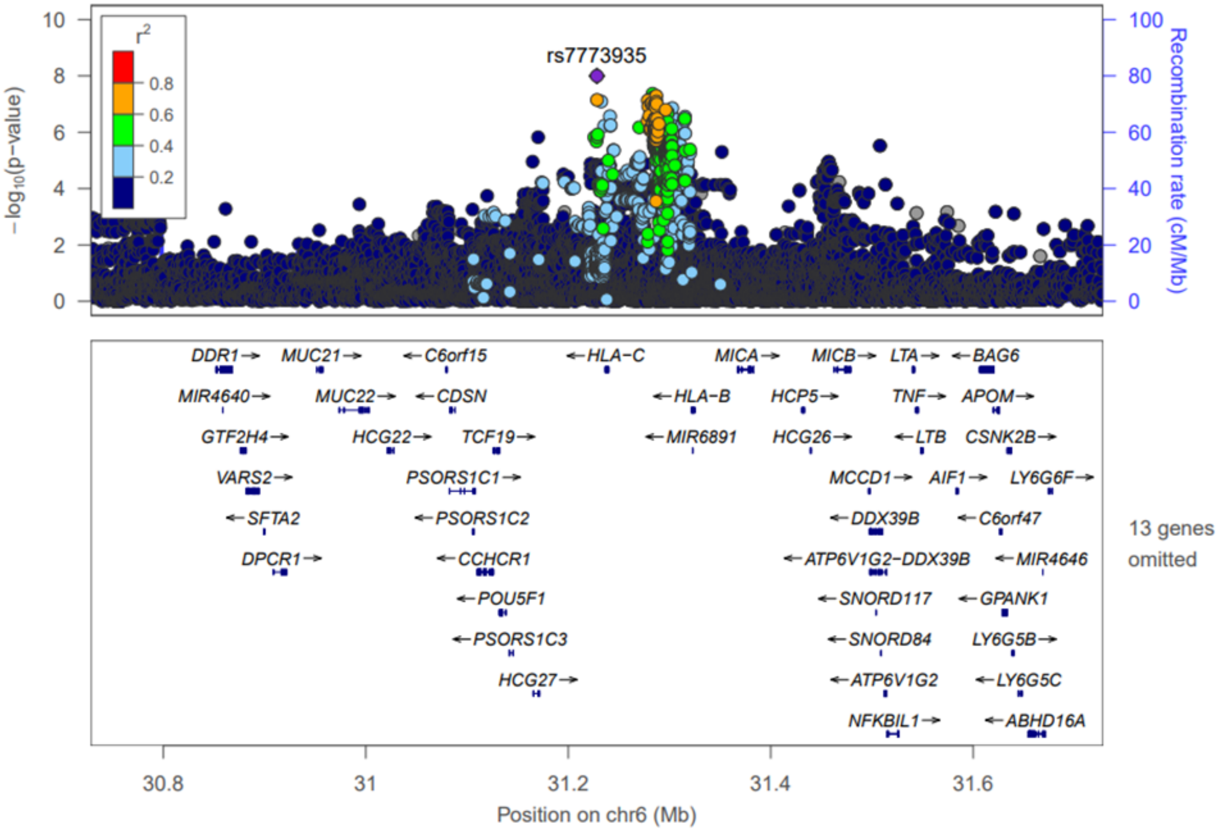

Supplementary Figure 5.7 LocusZoom plot for rs12460541 from GWAS for PC1

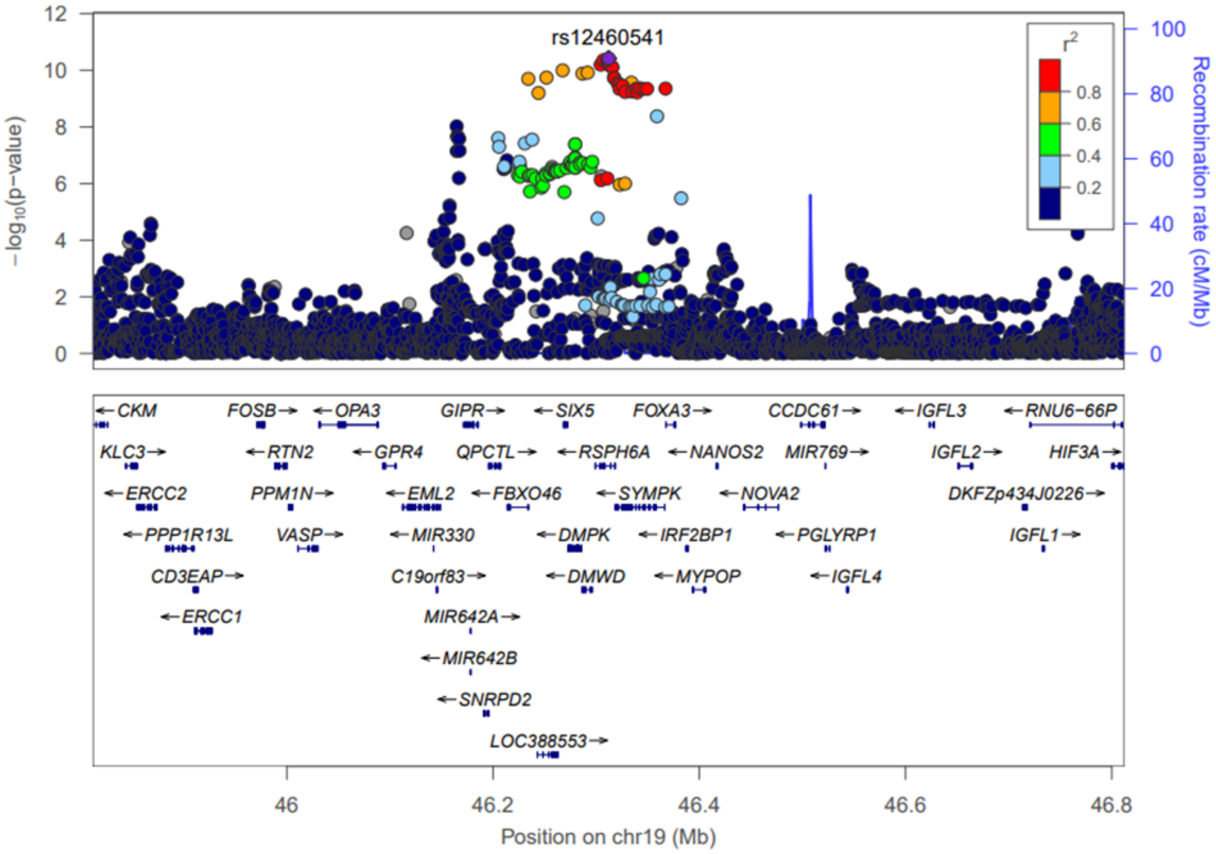

Supplementary Figure 5.8 LocusZoom plot for rs9886216 from GWAS for PC2

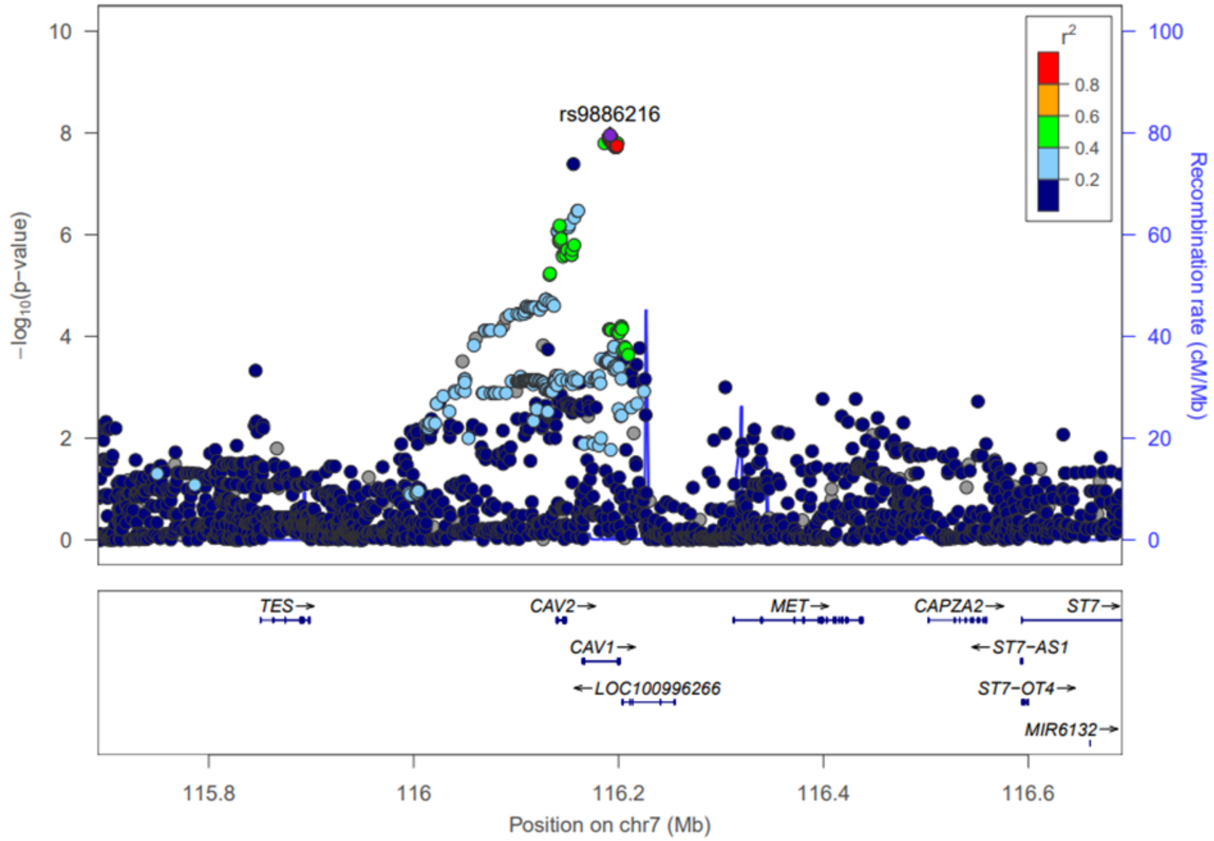

Supplementary Figure 5.9 LocusZoom plot for rs10155248 from GWAS for PC2

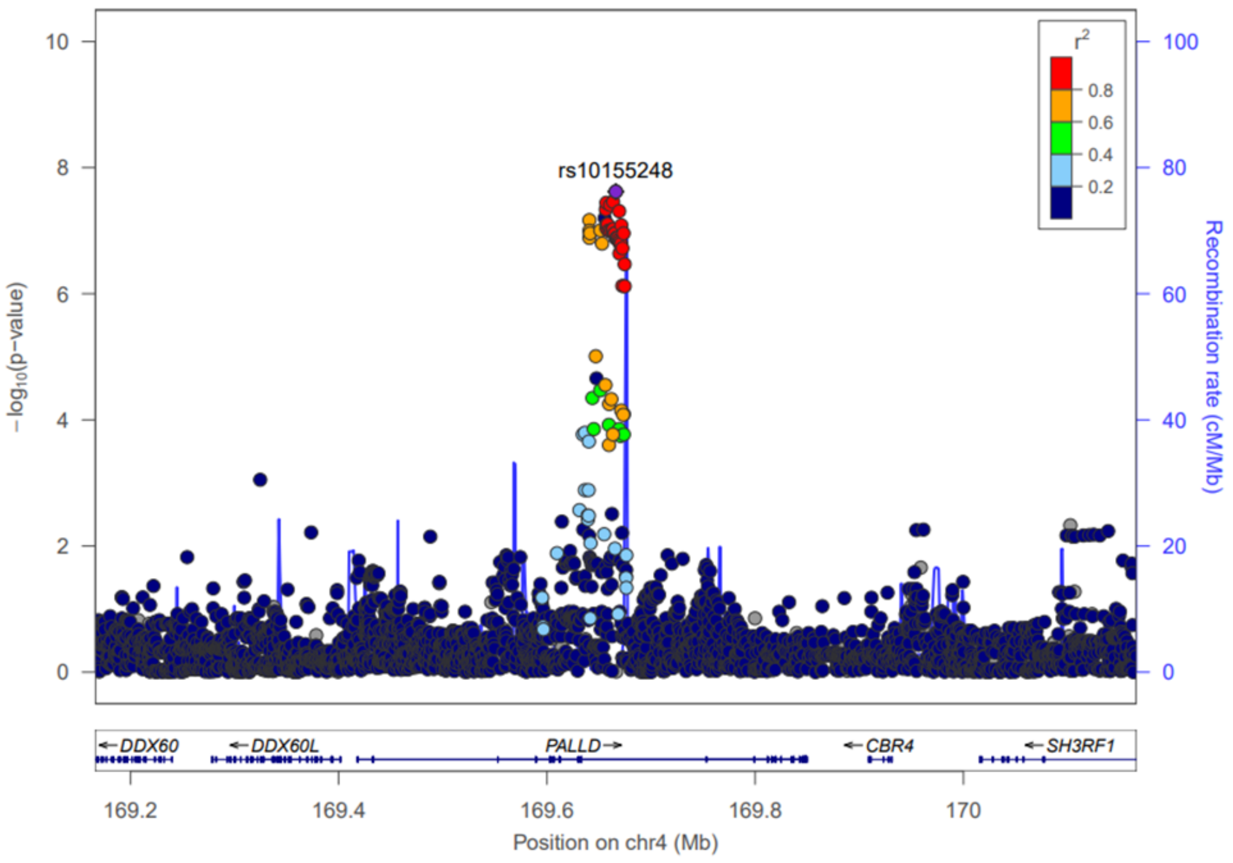

Supplementary Figure 5.10 LocusZoom plot for rs34723366 from GWAS for PC2

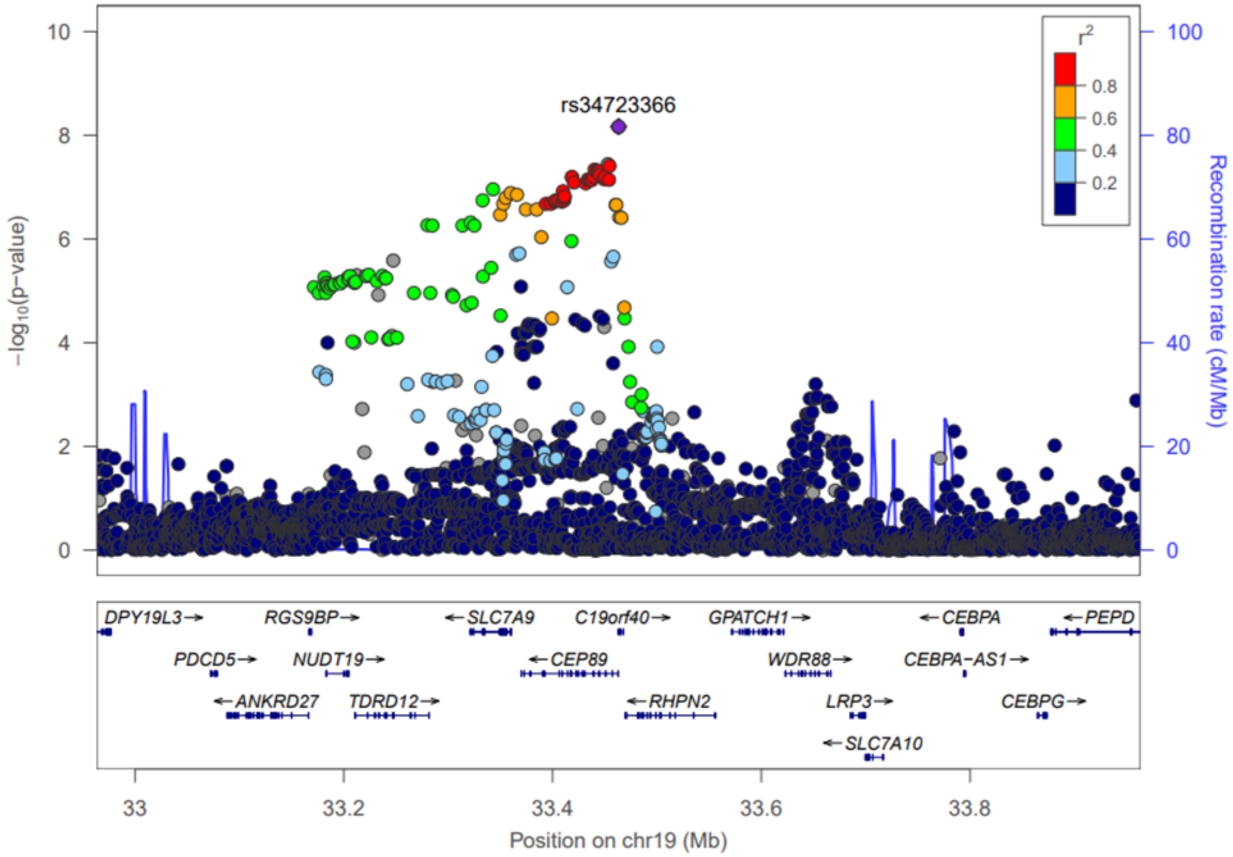

Supplementary Figure 5.11 LocusZoom plot for rs117953218 from GWAS for PC2

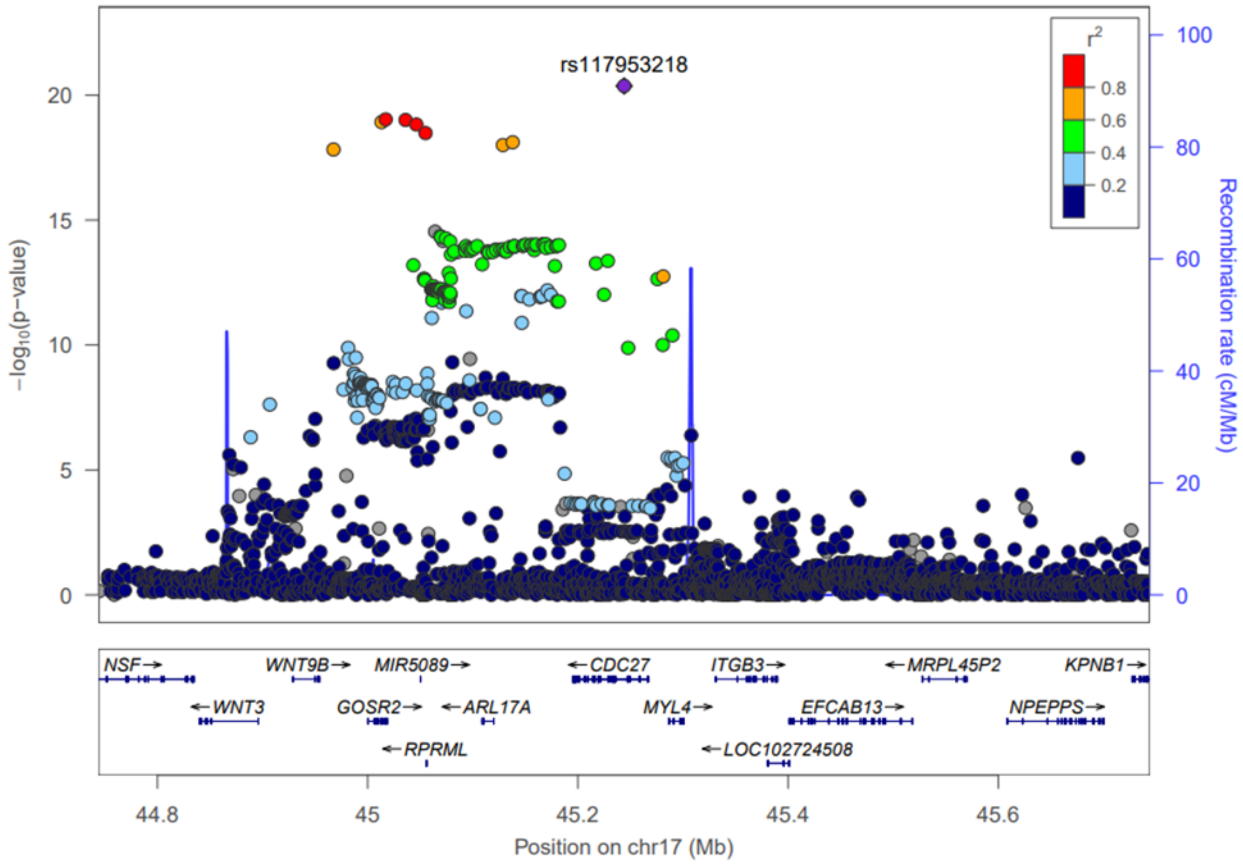

Supplementary Figure 5.12 LocusZoom plot for rs72706163 from GWAS for PC3

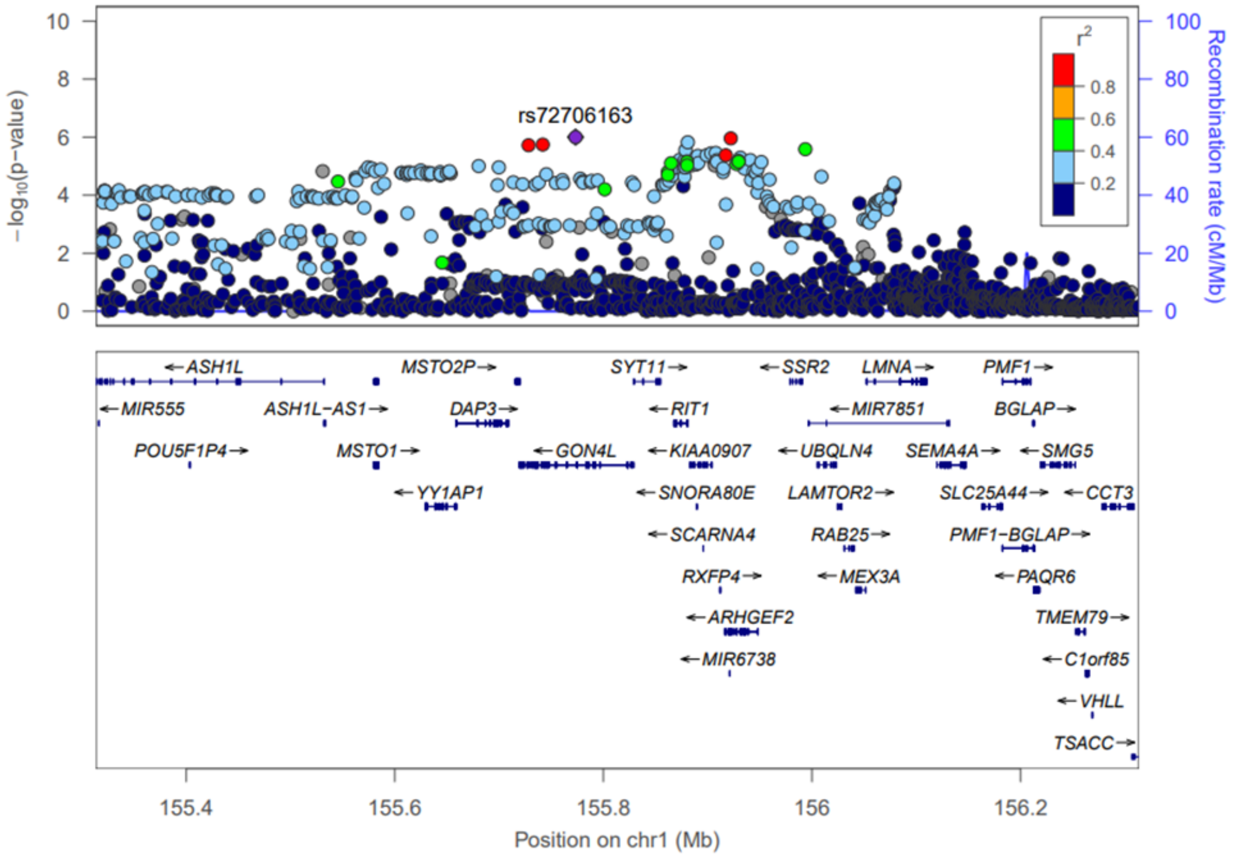

Supplementary Figure 5.13 LocusZoom plot for rs115456212 from GWAS for PC3

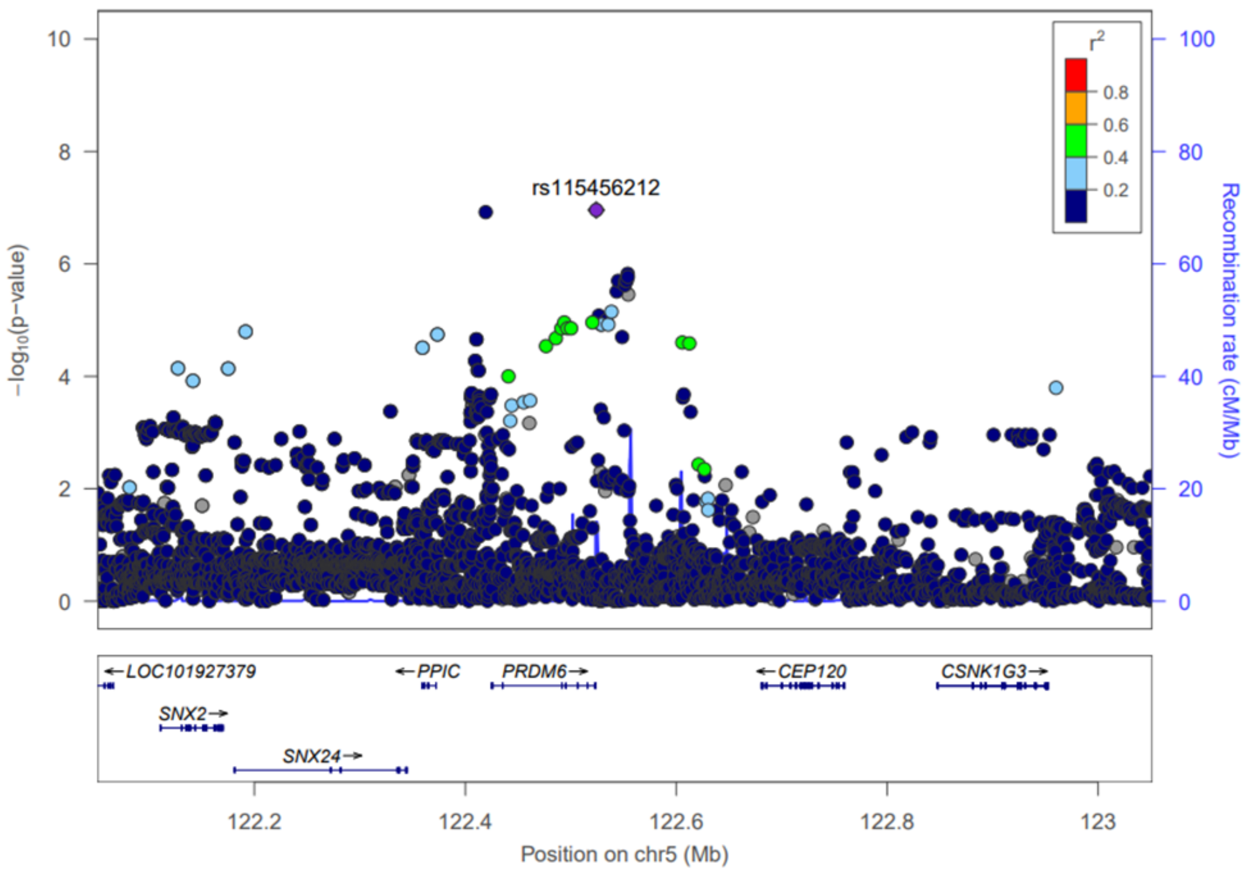

Supplementary Figure 5.14 LocusZoom plot for rs452036 from GWAS for PC3

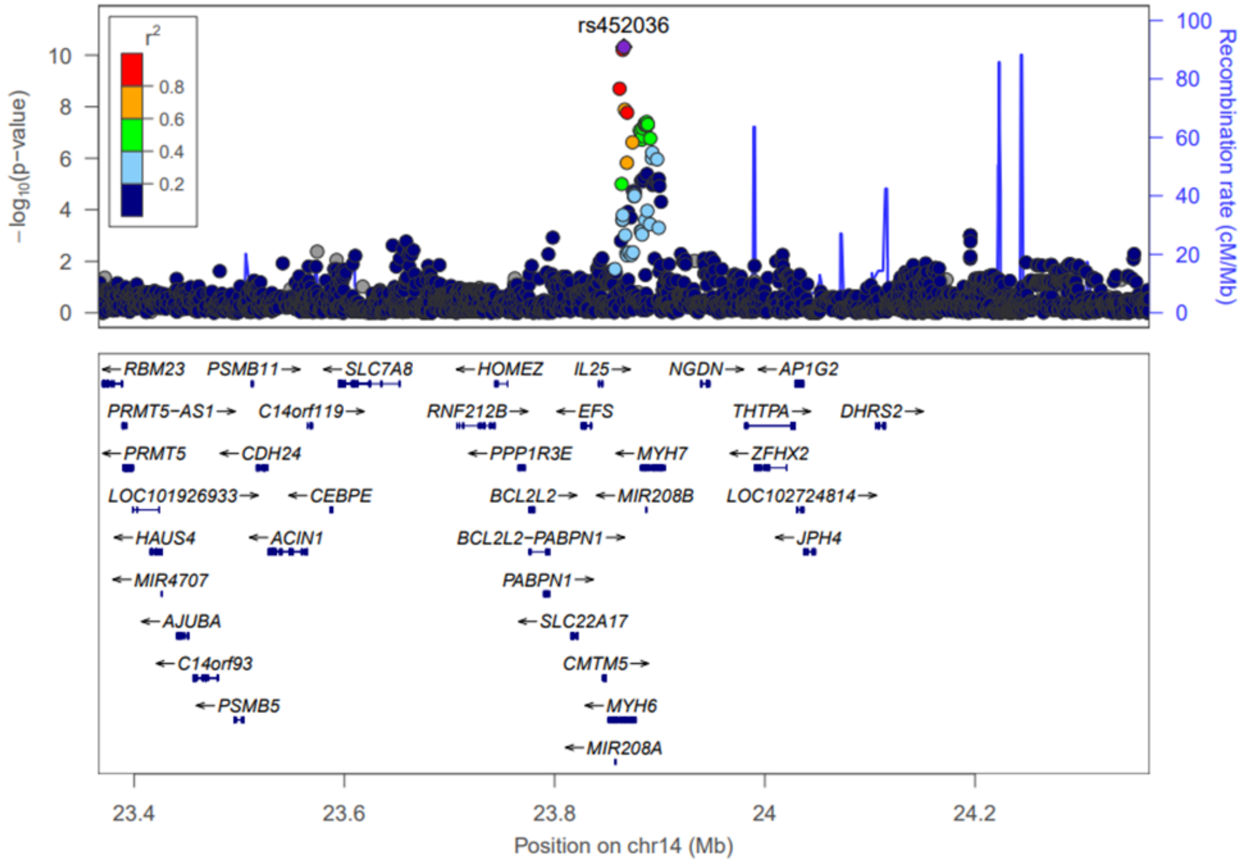

Supplementary Figure 5.15 LocusZoom plot for rs3176326 from GWAS for PC3

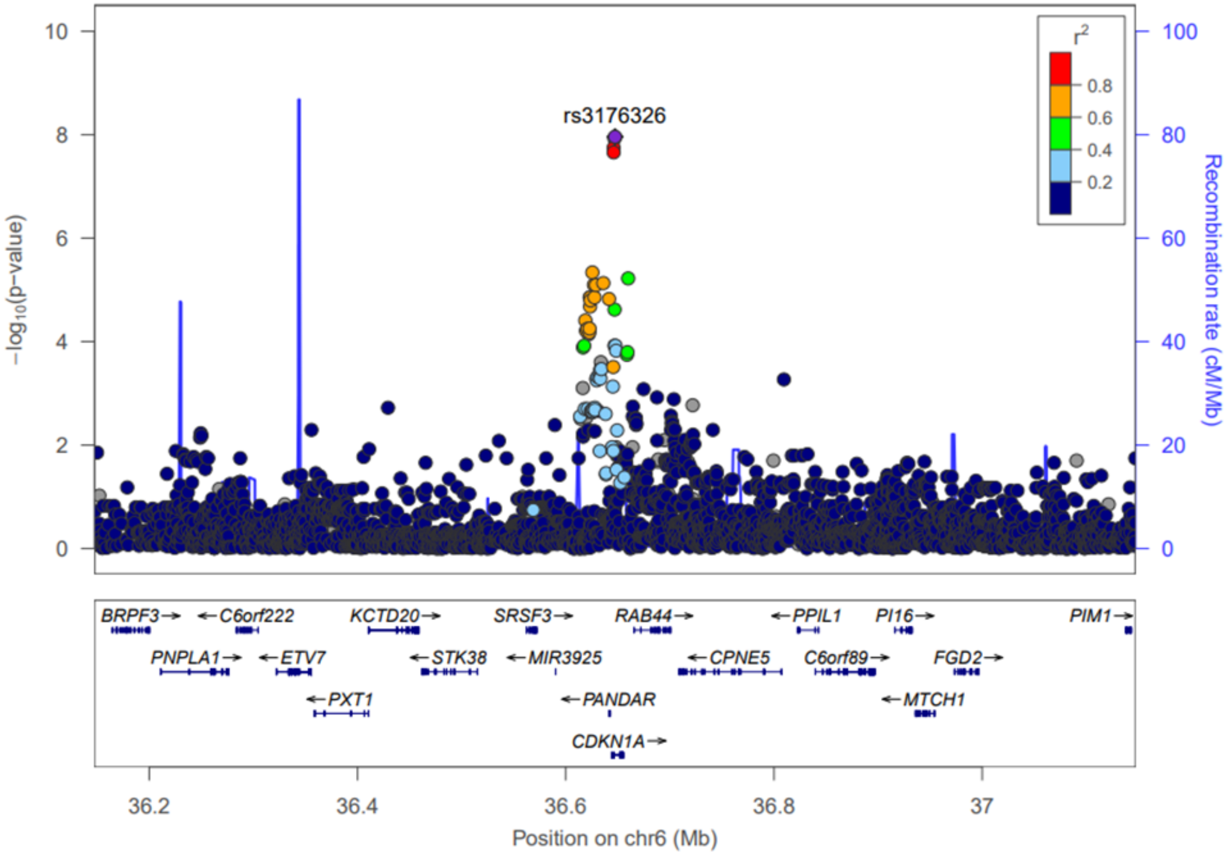

Supplementary Figure 5.16 LocusZoom plot for rs10950714 from GWAS for PC3

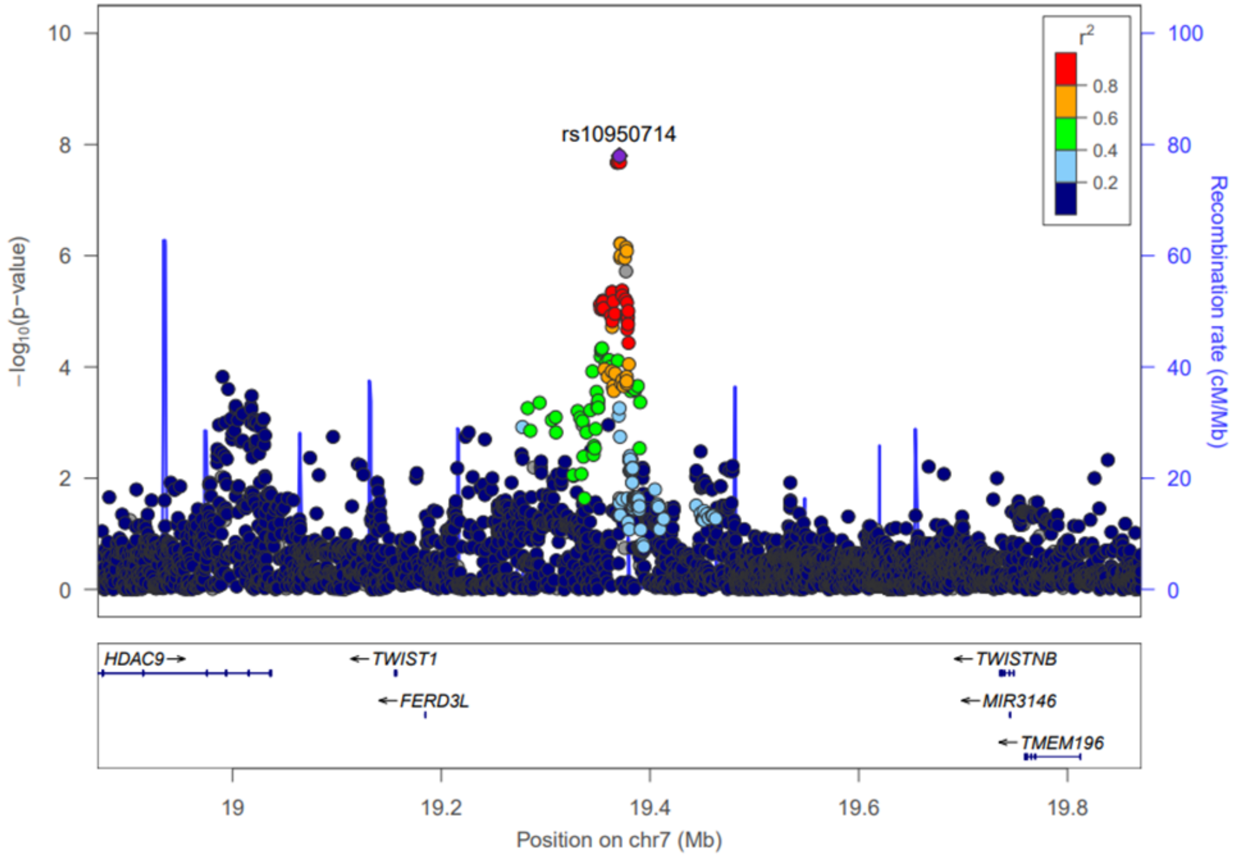

Supplementary Figure 5.17 LocusZoom plot for rs11238134 from GWAS for PC3

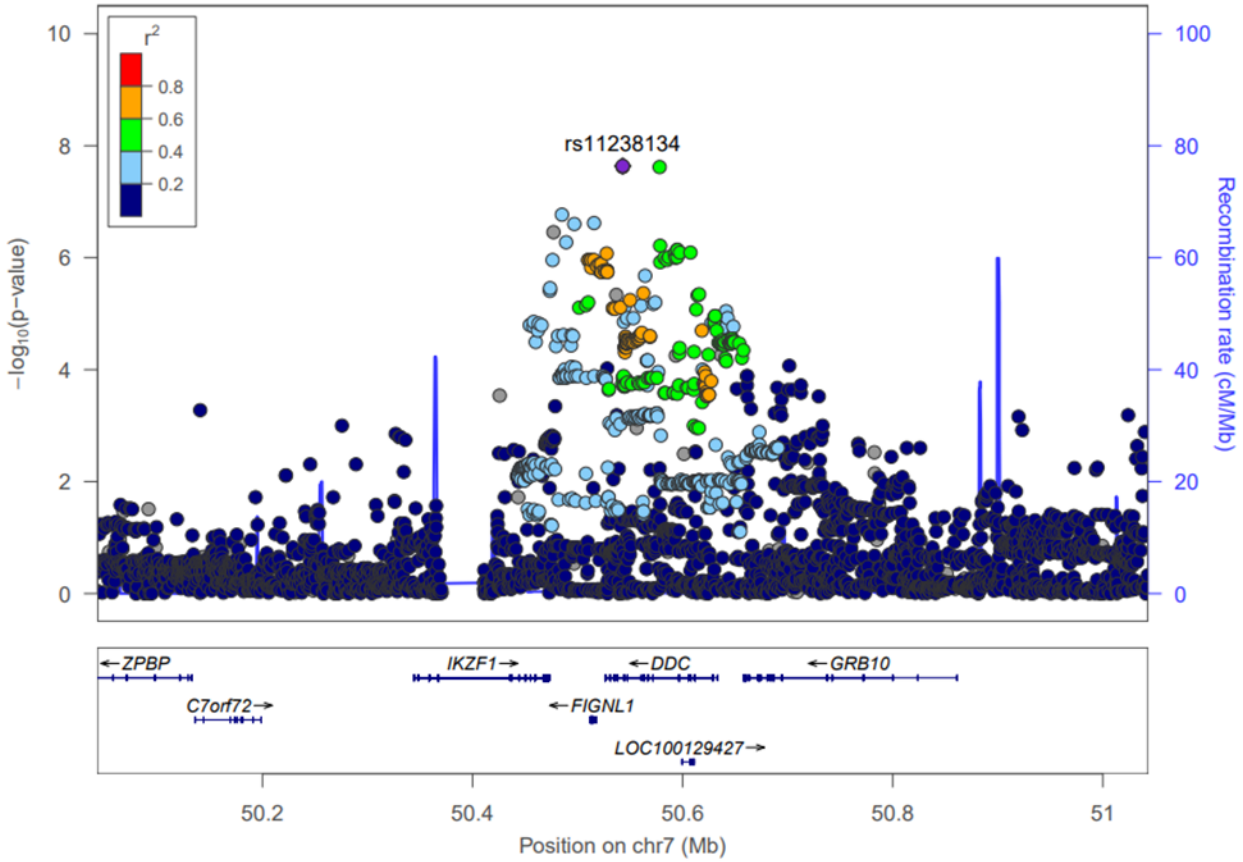

Supplementary Figure 5.18 LocusZoom plot for rs34092536 from GWAS for PC4

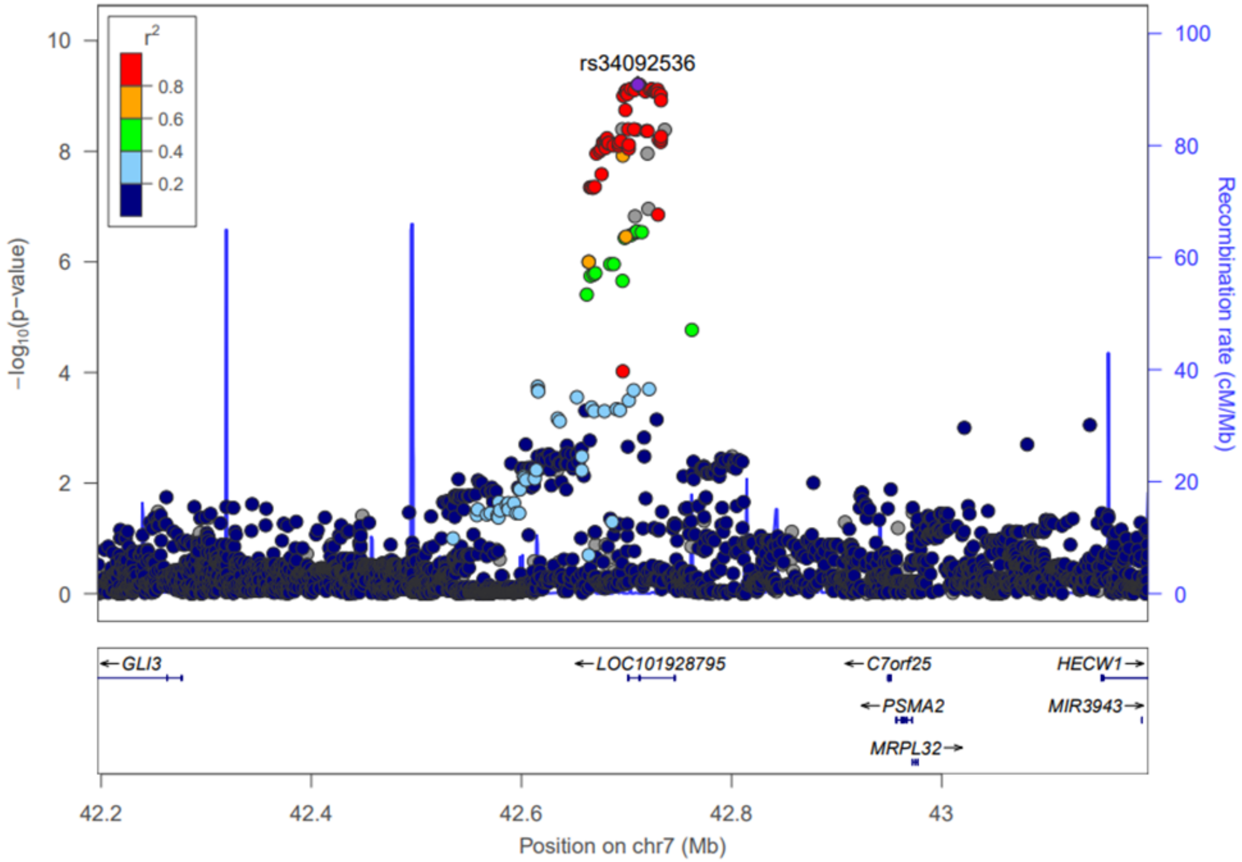

Supplementary Figure 5.19 LocusZoom plot for rs143823594 from GWAS for PC4

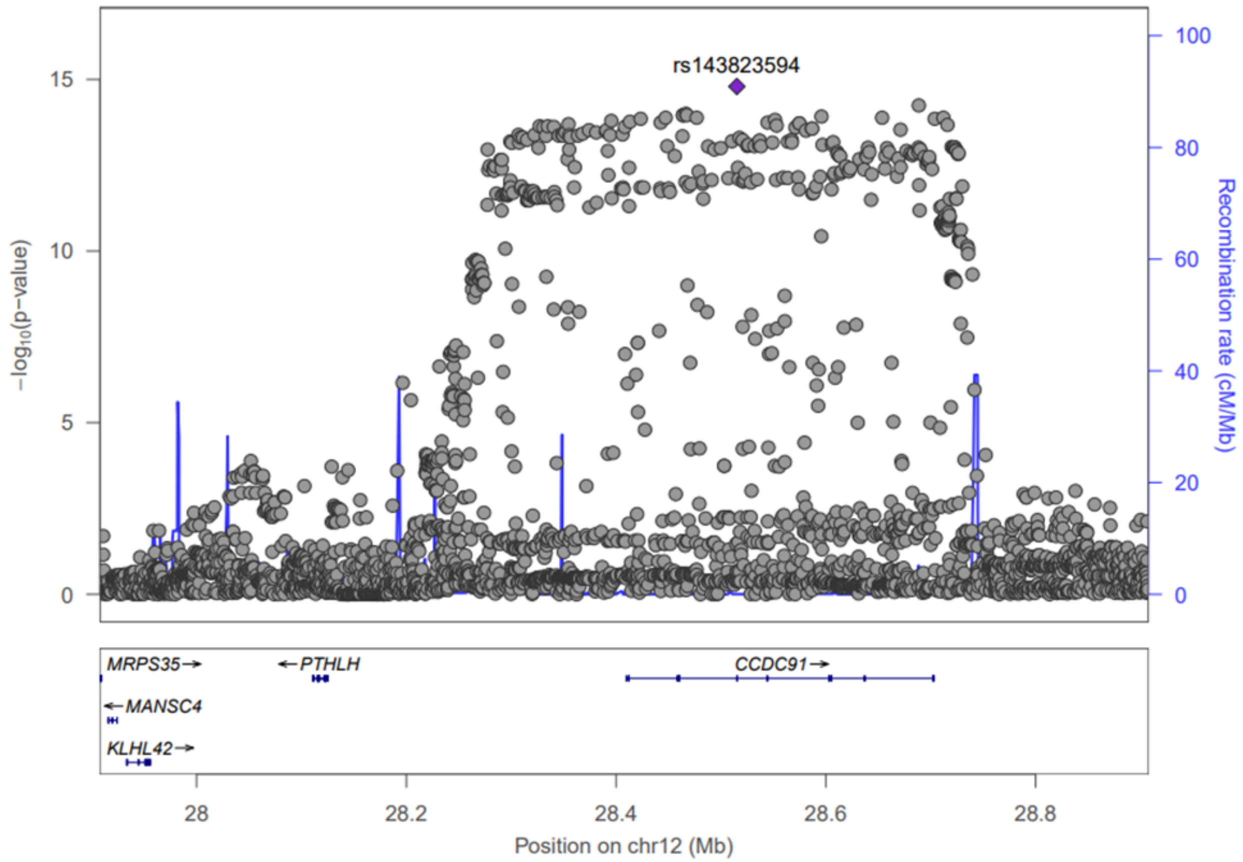

Supplementary Figure 5.20 LocusZoom plot for rs3801387 from GWAS for PC4

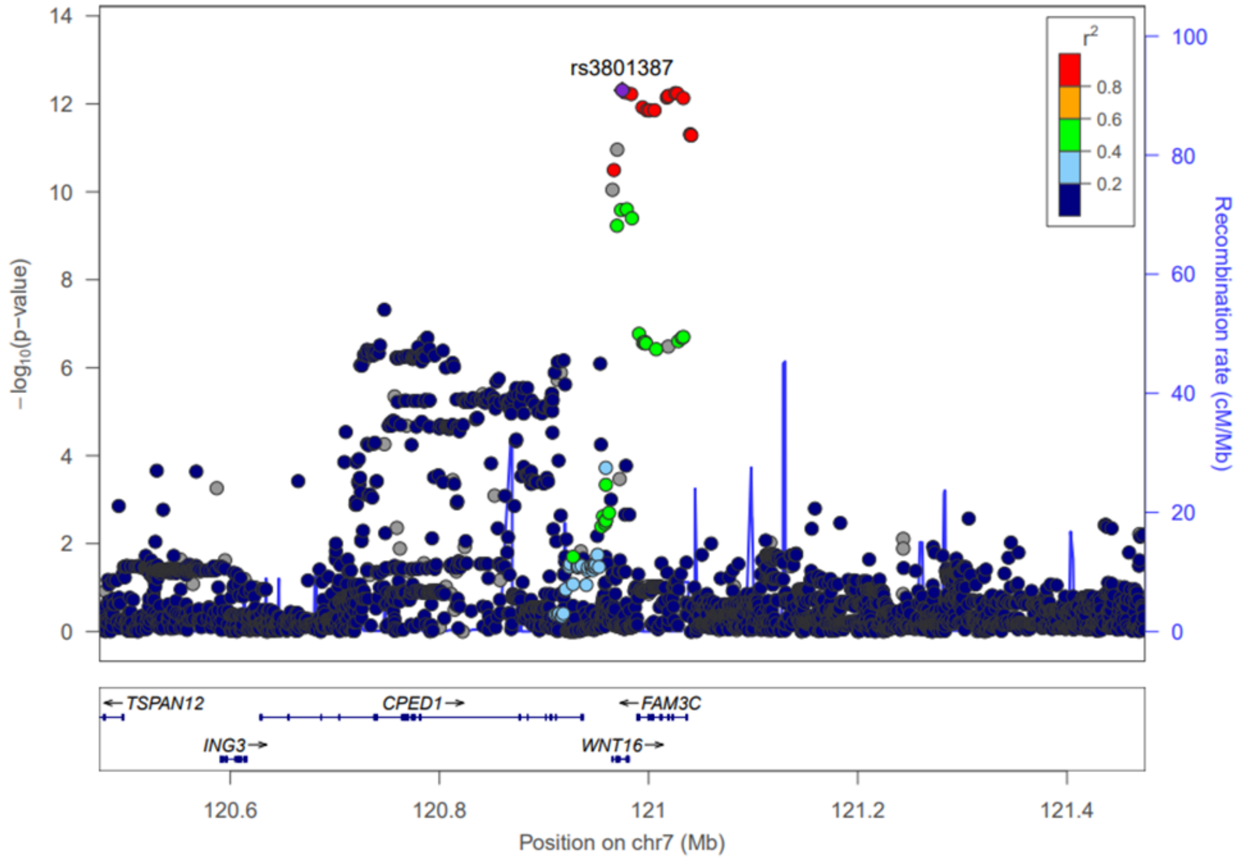

Supplementary Figure 5.21 LocusZoom plot for rs4414033 from GWAS for PC4

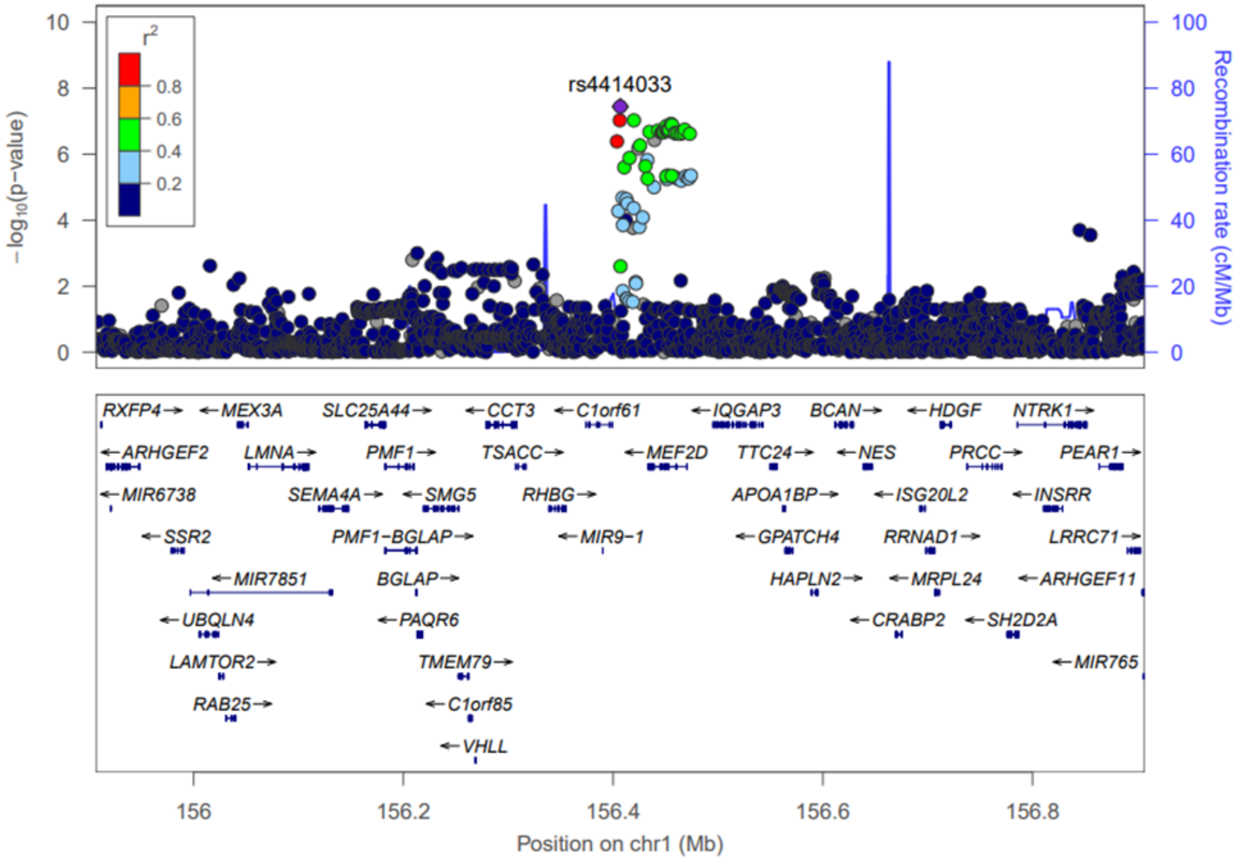

Supplementary Figure 5.22 LocusZoom plot for rs4466228 from GWAS for PC4

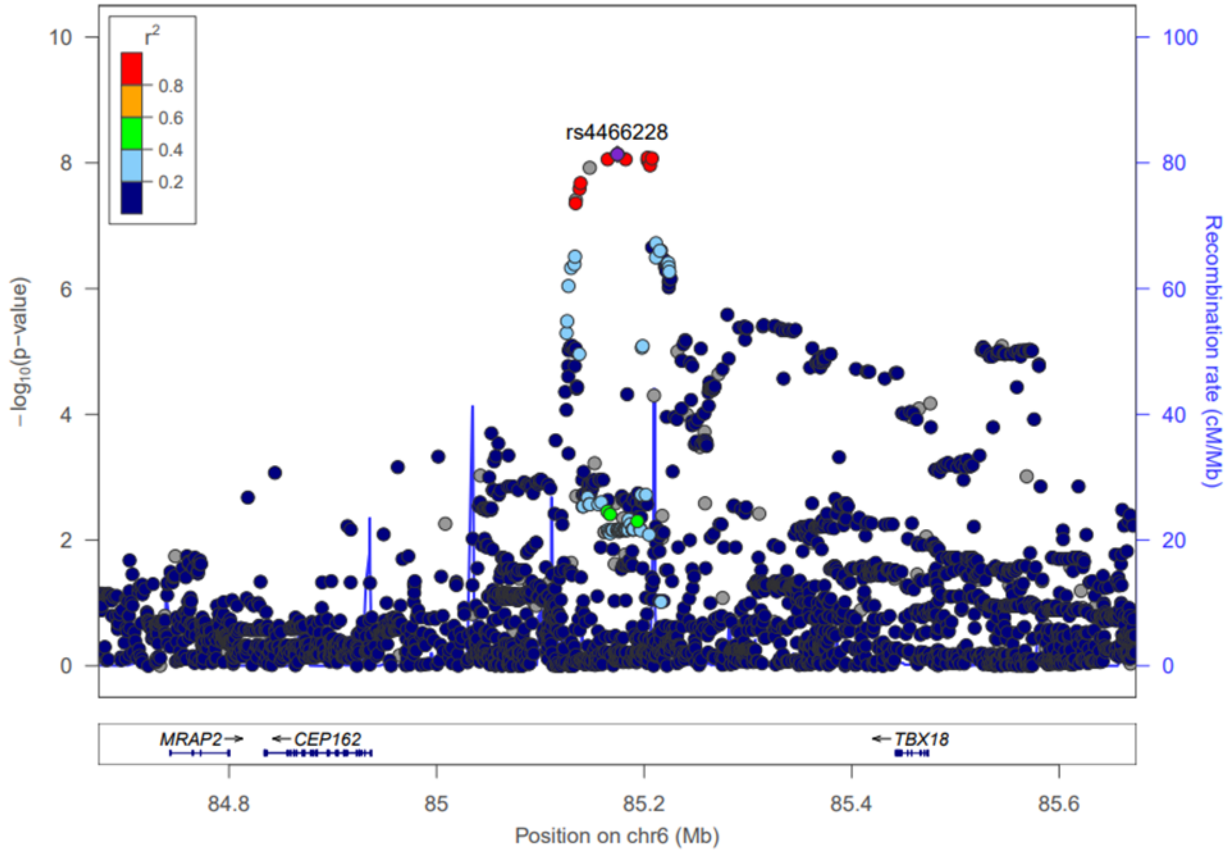

Supplementary Figure 5.23 LocusZoom plot for rs4937553 from GWAS for PC4

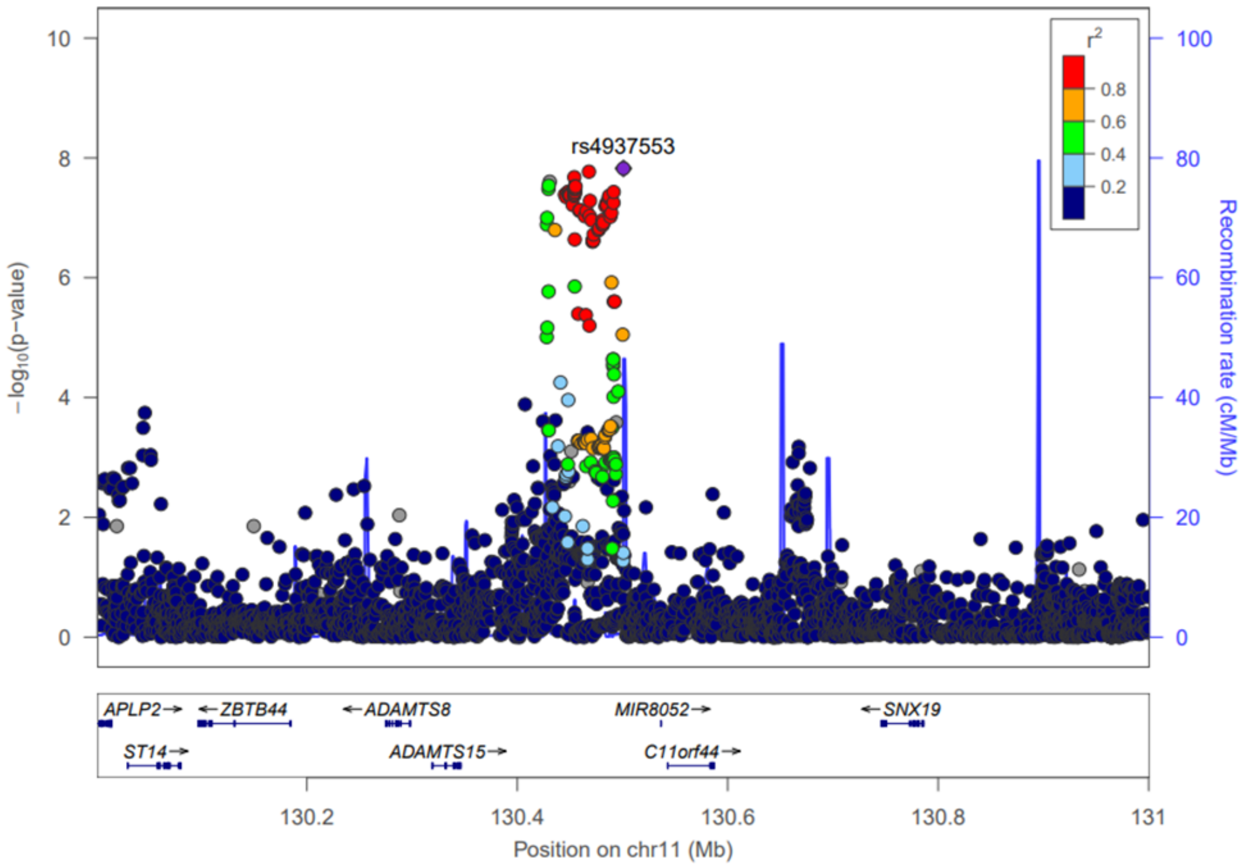

Supplementary Figure 5.24 LocusZoom plot for rs7620927 from GWAS for PC4

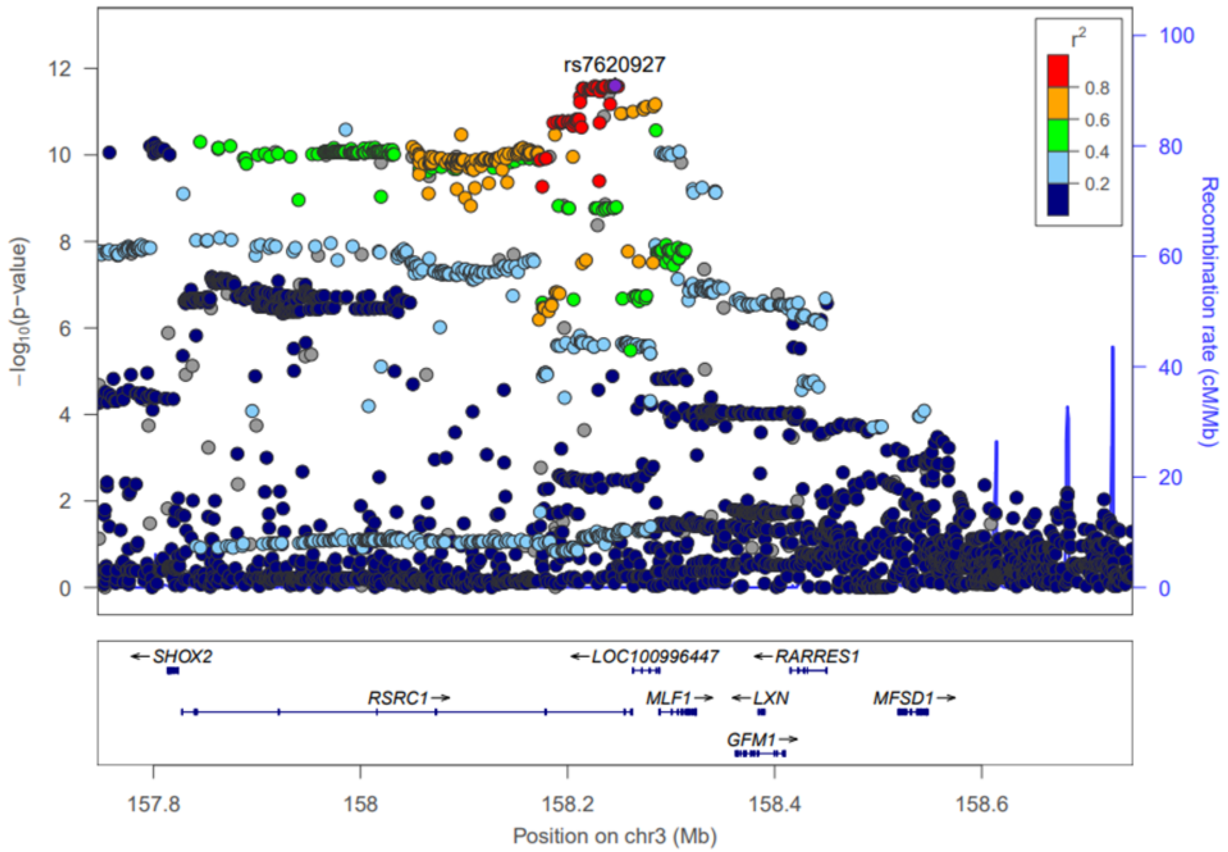

Supplementary Figure 5.25 LocusZoom plot for rs332101 from GWAS for PC5

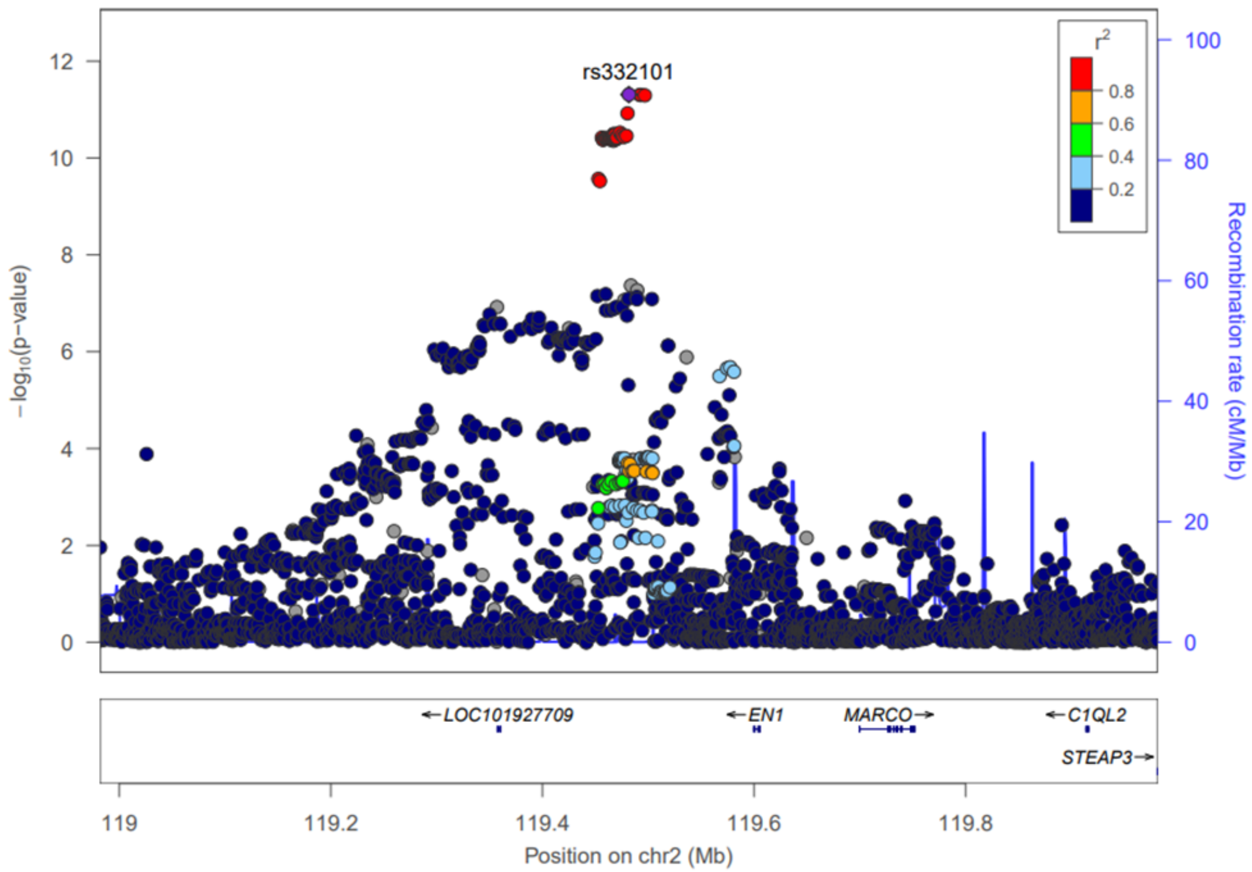

Supplementary Figure 5.26 LocusZoom plot for rs669738 from GWAS for PC5

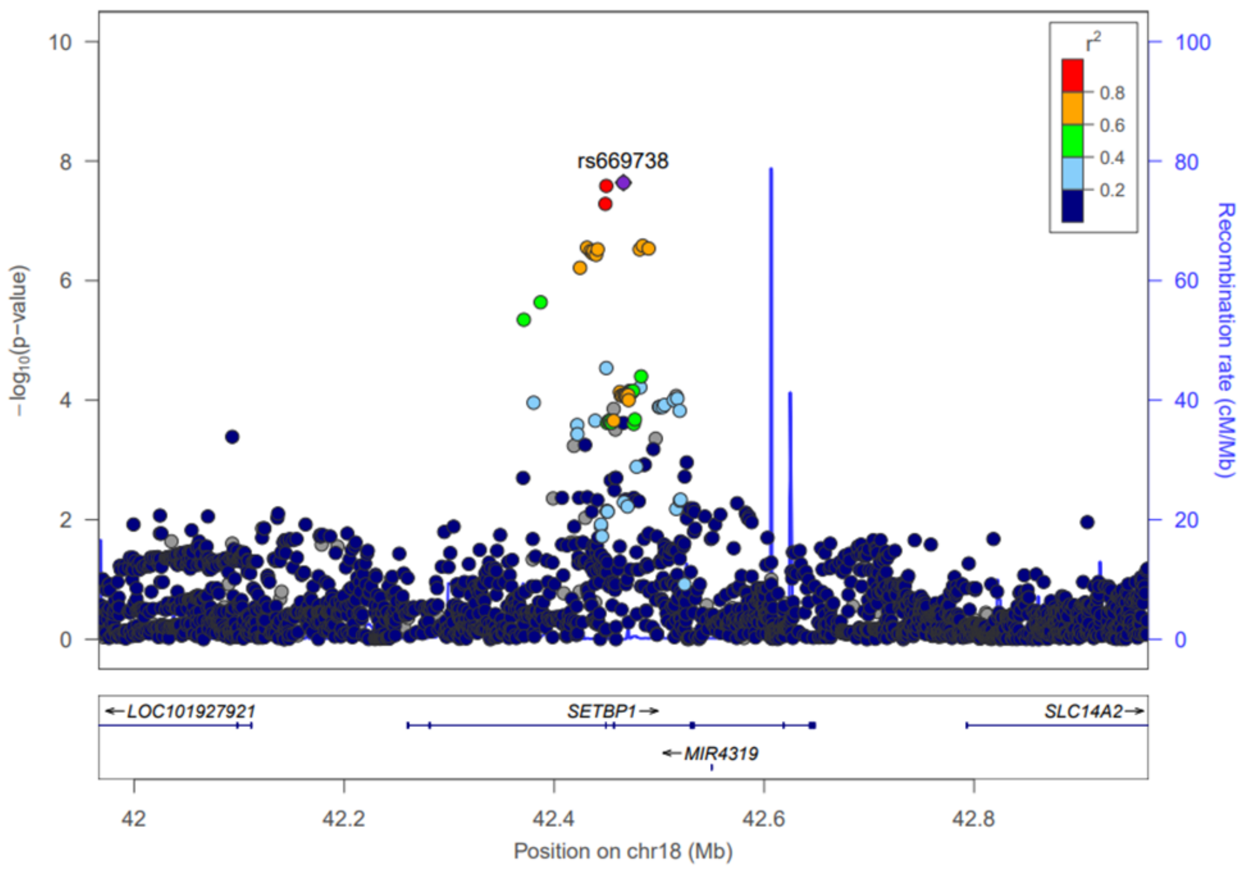

Supplementary Figure 5.27 LocusZoom plot for rs791274 from GWAS for PC5

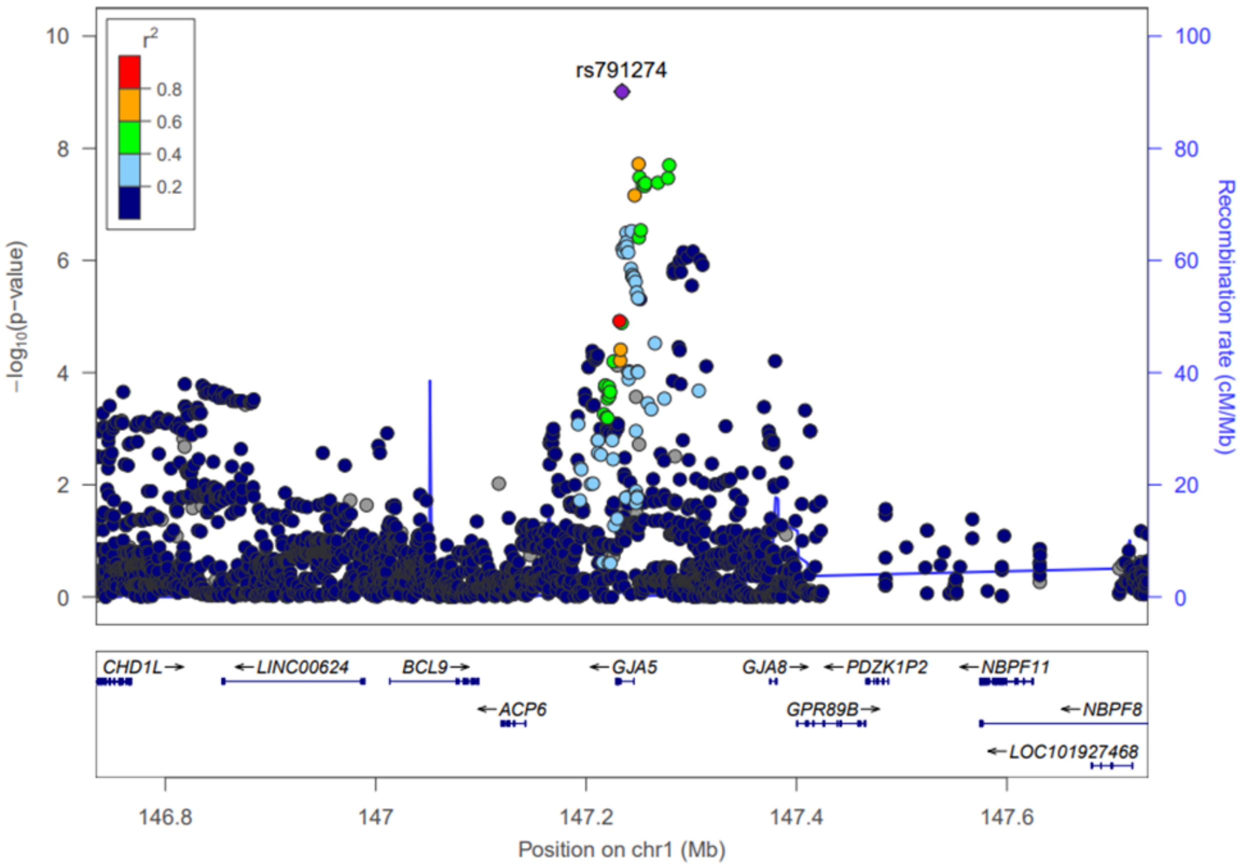

Supplementary Figure 5.28 LocusZoom plot for rs3951016 from GWAS for PC5

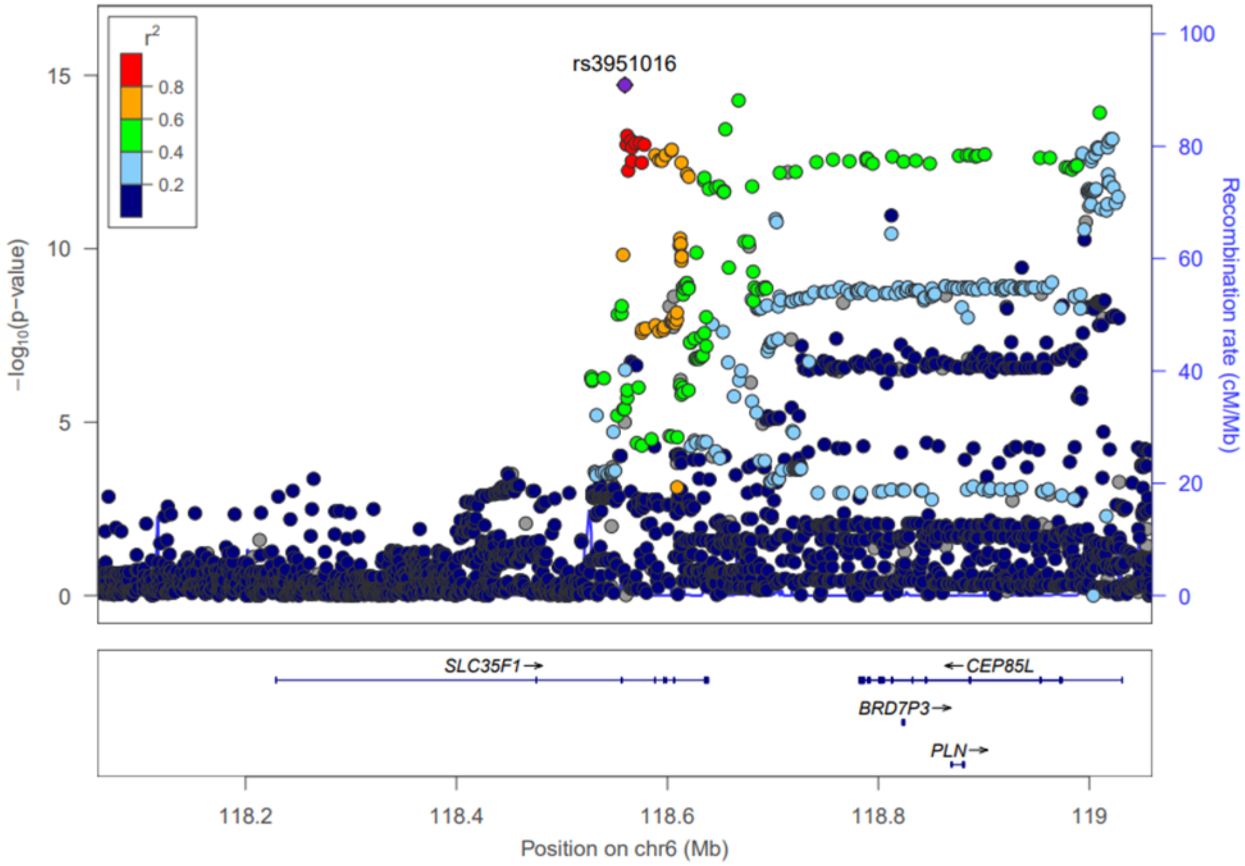

Supplementary Figure 5.29 LocusZoom plot for rs4659701 from GWAS for PC5

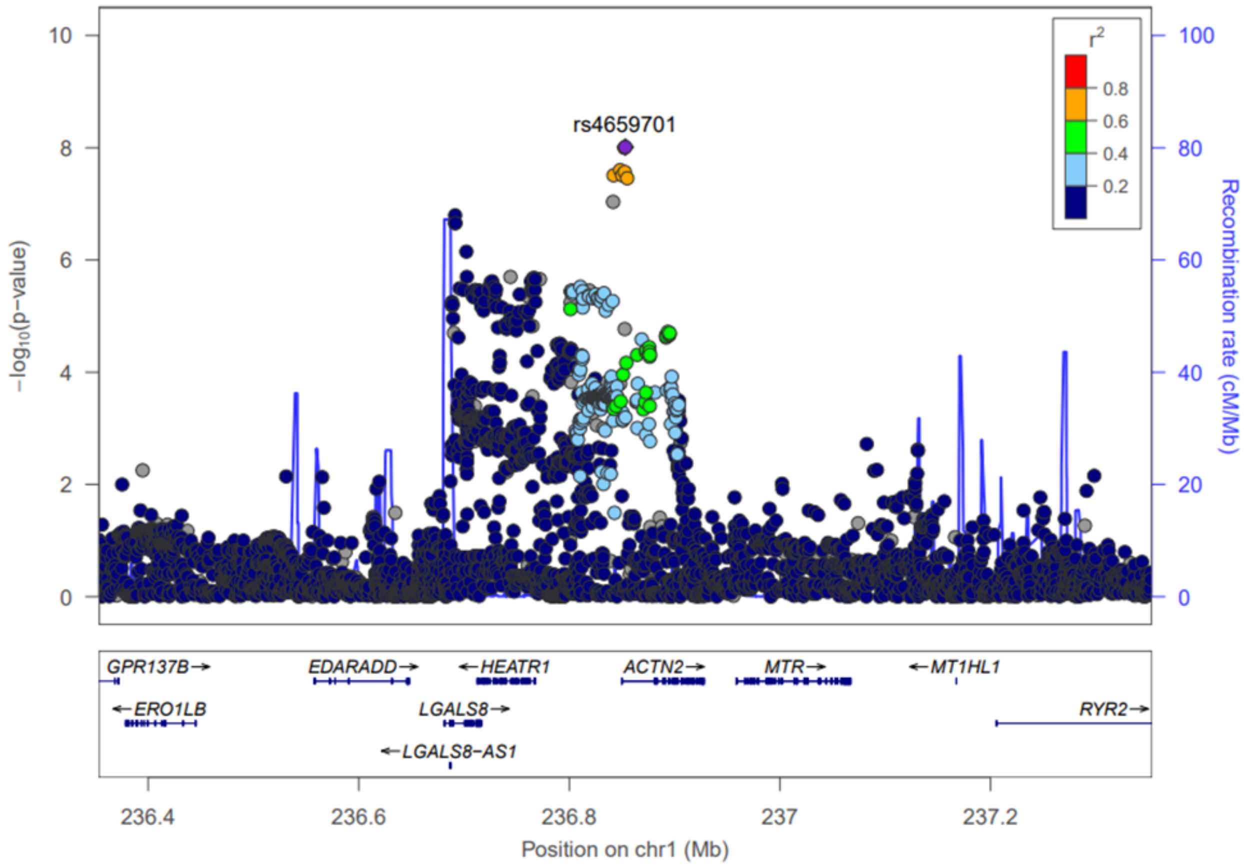

Supplementary Figure 5.30 LocusZoom plot for rs10155223 from GWAS for PC5

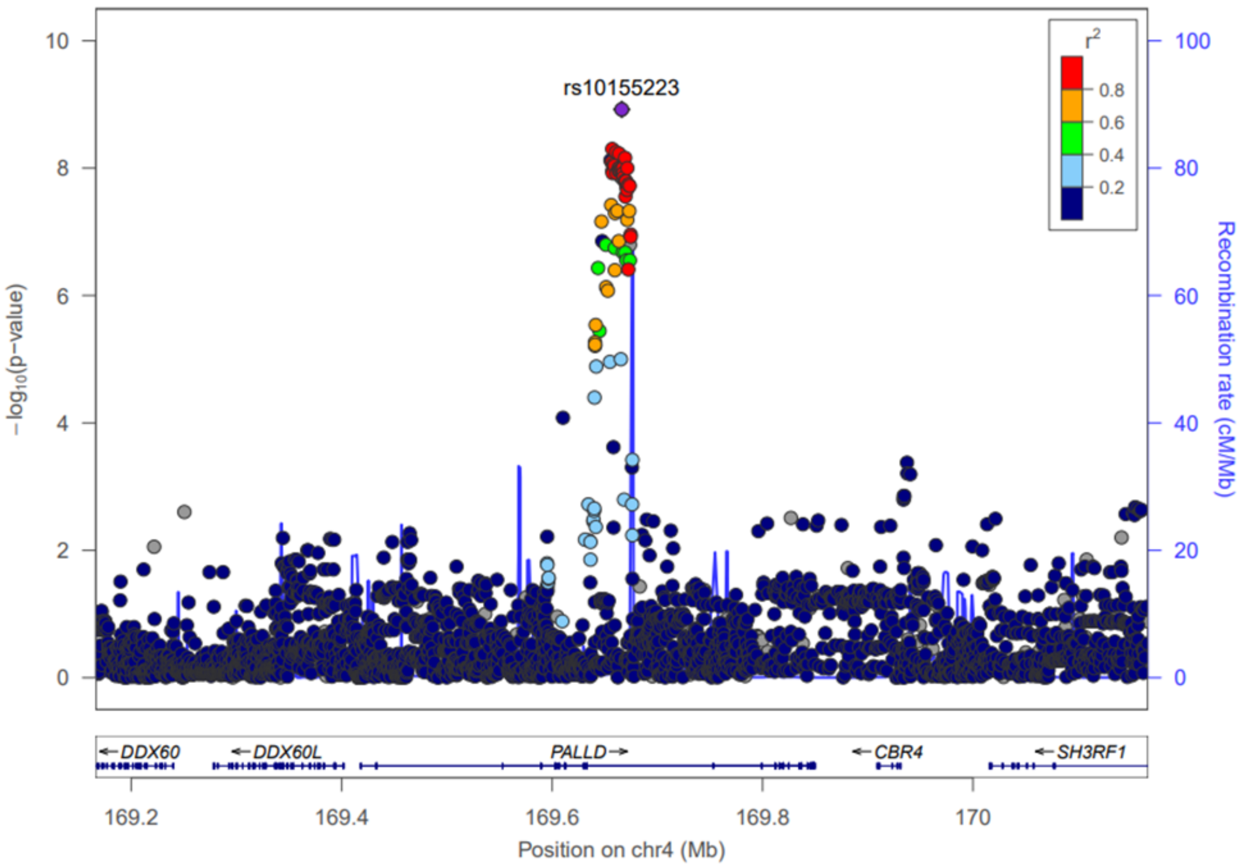

Supplementary Figure 5.31 LocusZoom plot for rs10193295 from GWAS for PC5

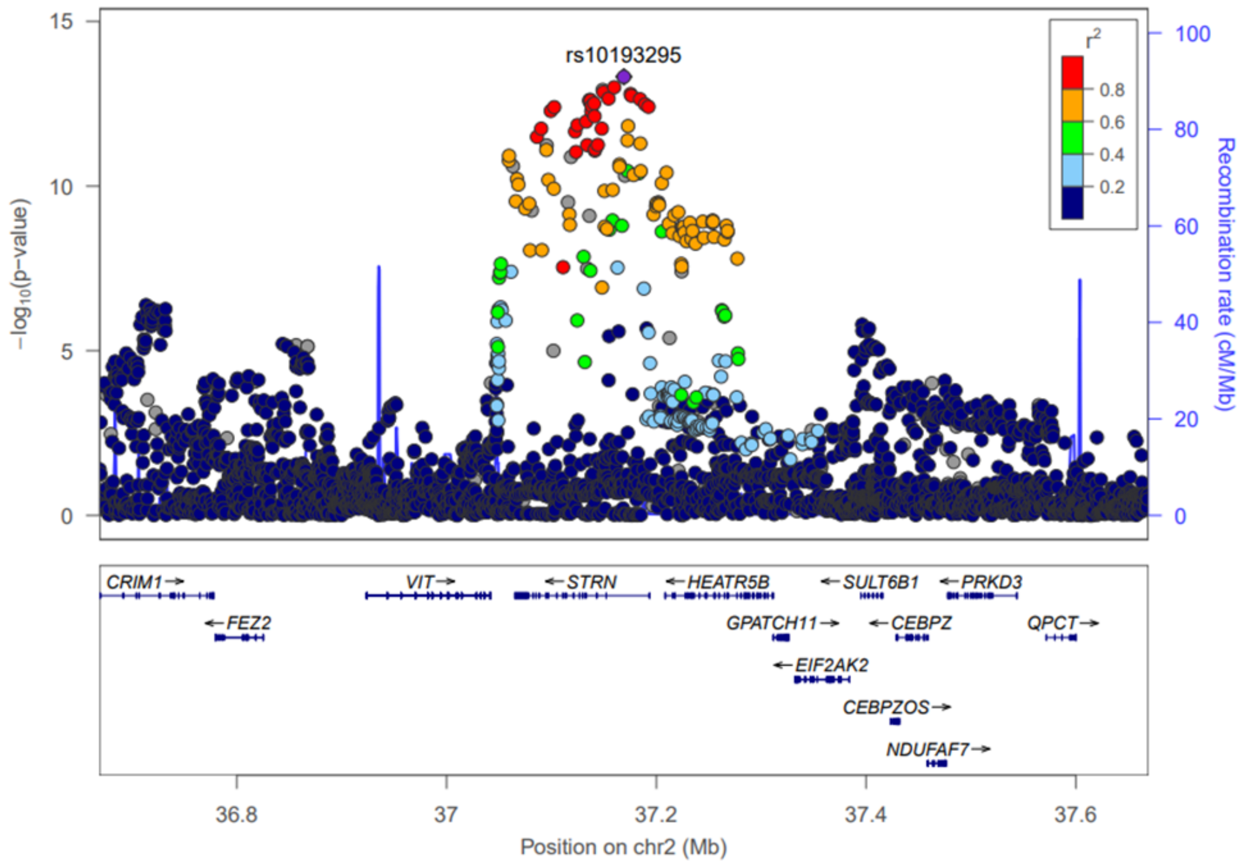

Supplementary Figure 5.32 LocusZoom plot for rs57655466 from GWAS for PC5

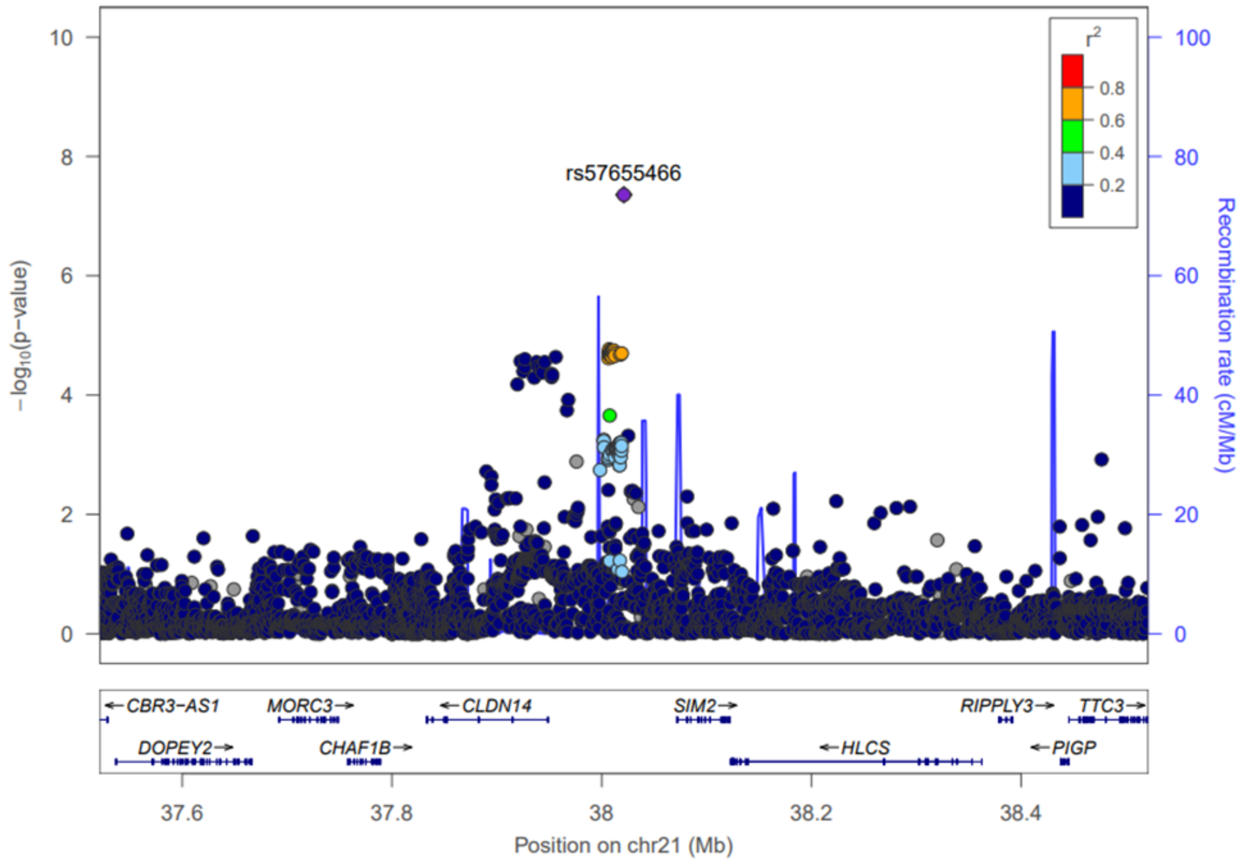

Supplementary Figure 5.33 LocusZoom plot for rs146170154 from GWAS for PC5

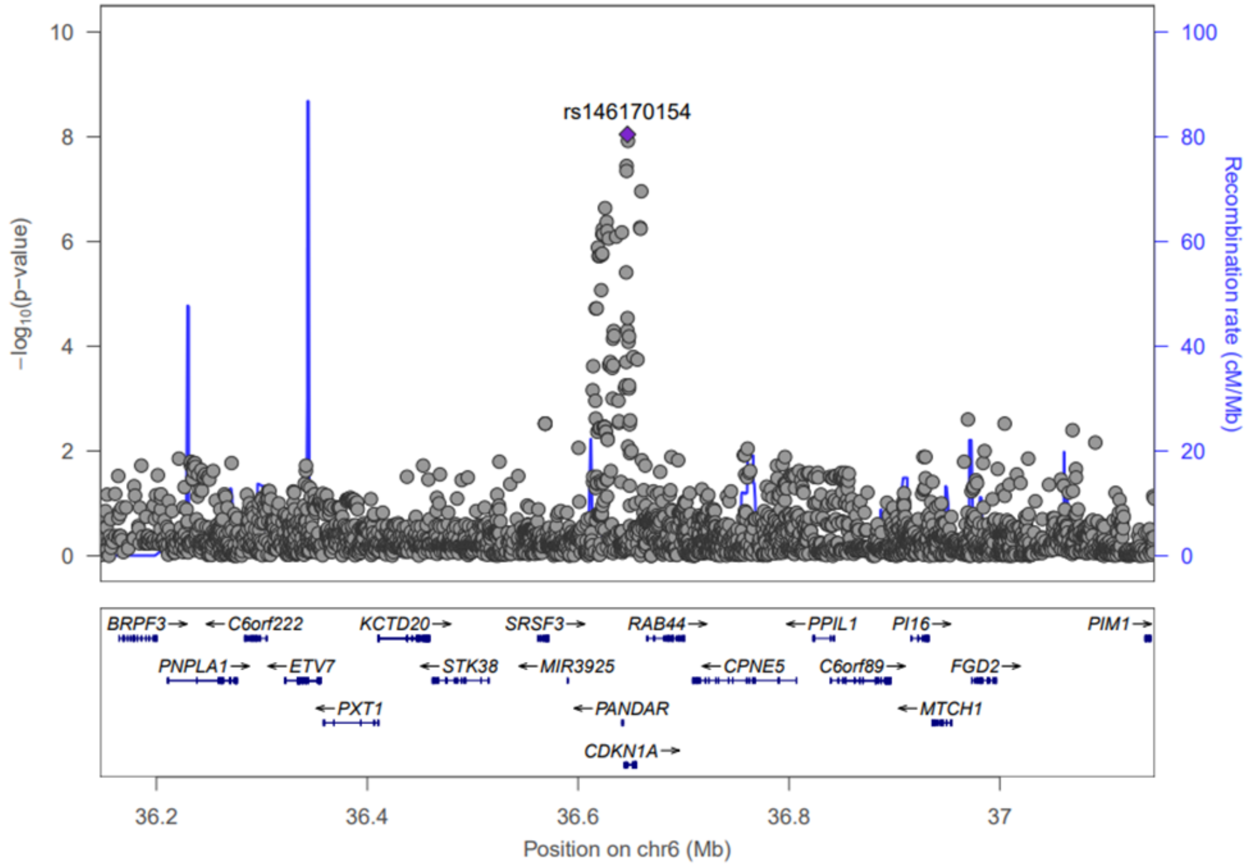

Supplementary Figure 5.34 LocusZoom plot for rs11703407 from GWAS for PC8

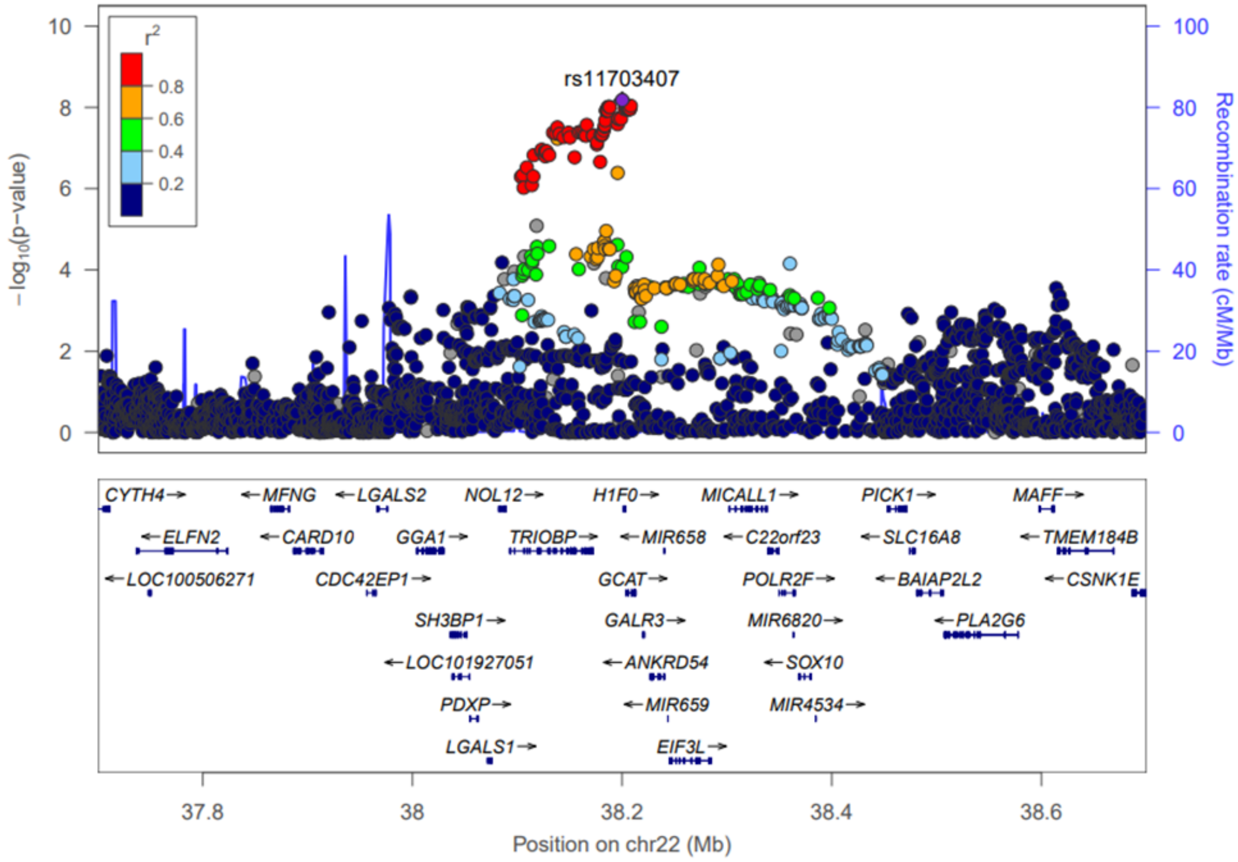

Supplementary Figure 5.35 LocusZoom plot for rs35001652 from GWAS for PC8

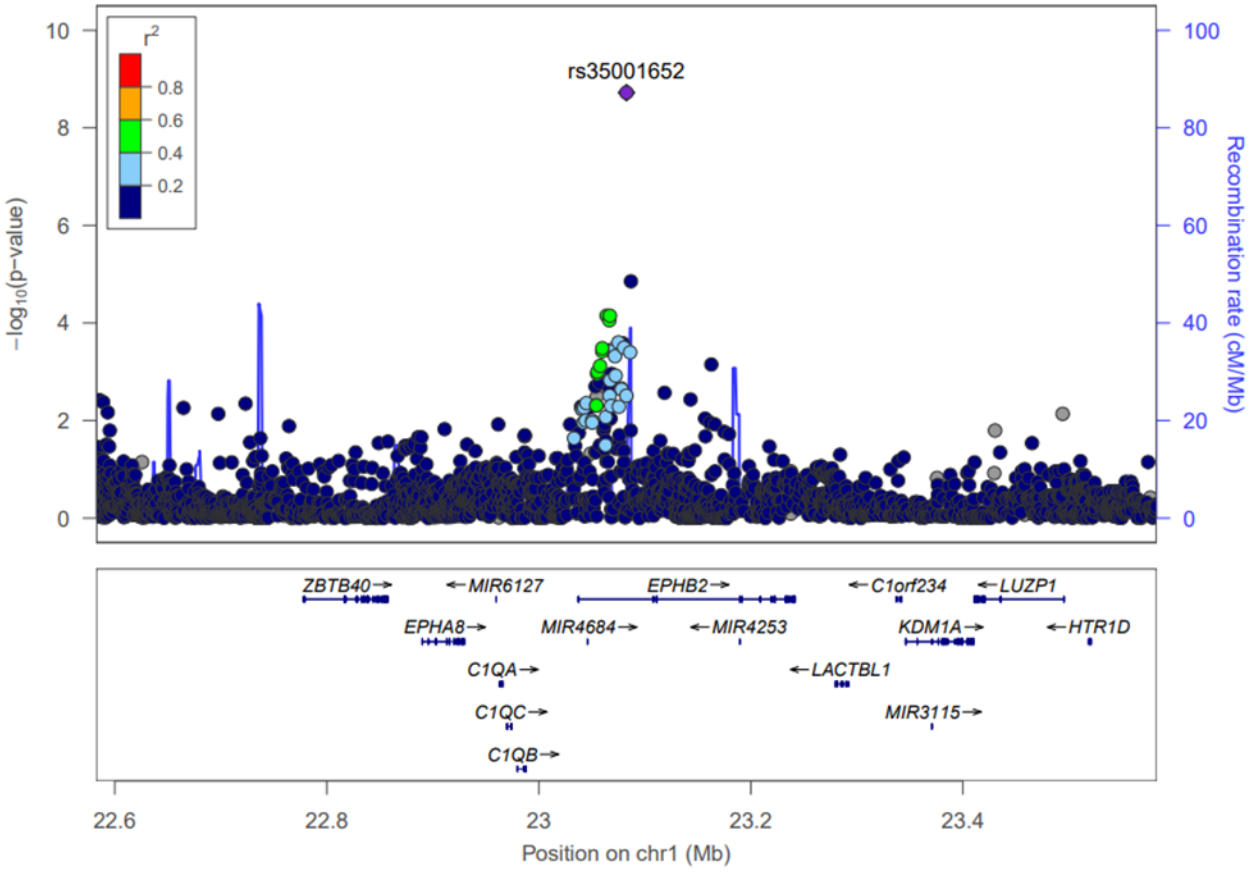

Supplementary Figure 5.36 LocusZoom plot for rs1461990 from GWAS for PC9

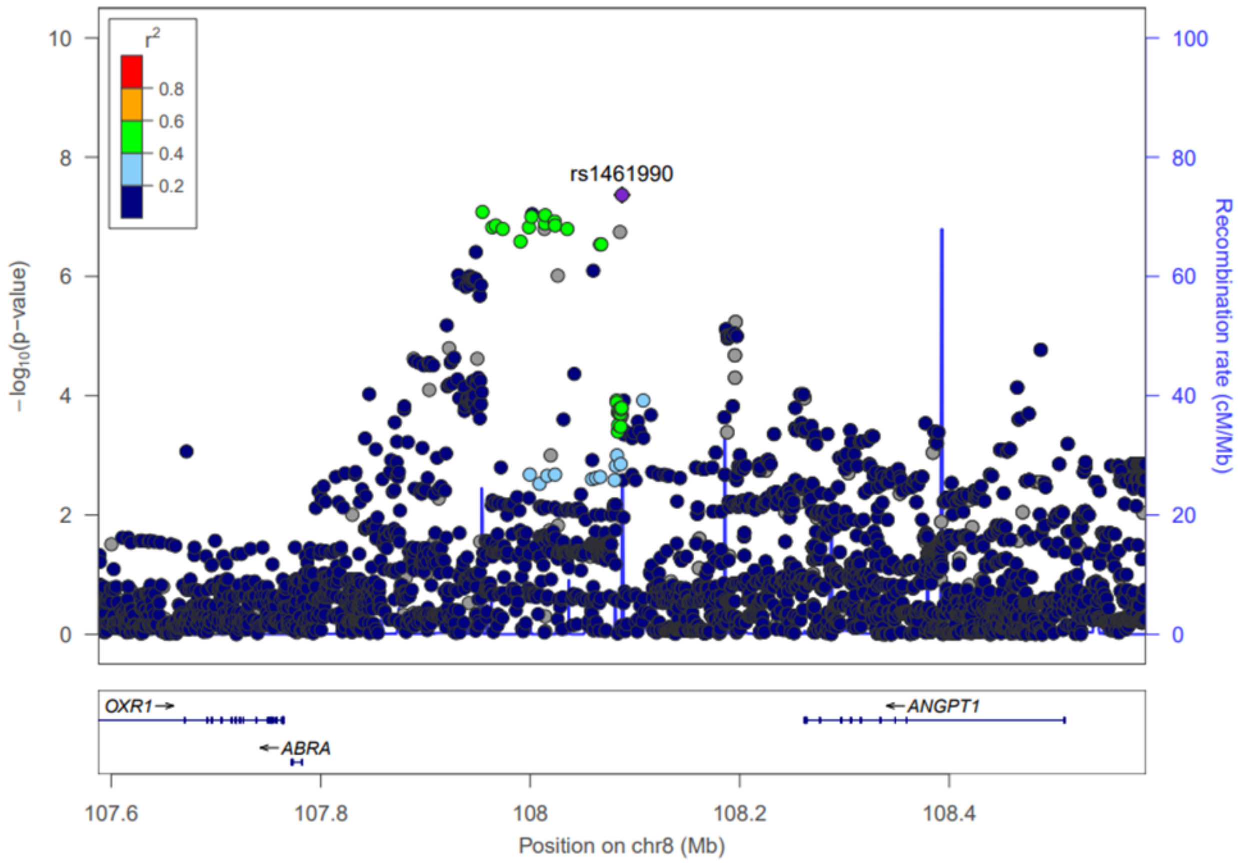

Supplementary Figure 5.37 LocusZoom plot for rs4836390 from GWAS for PC9

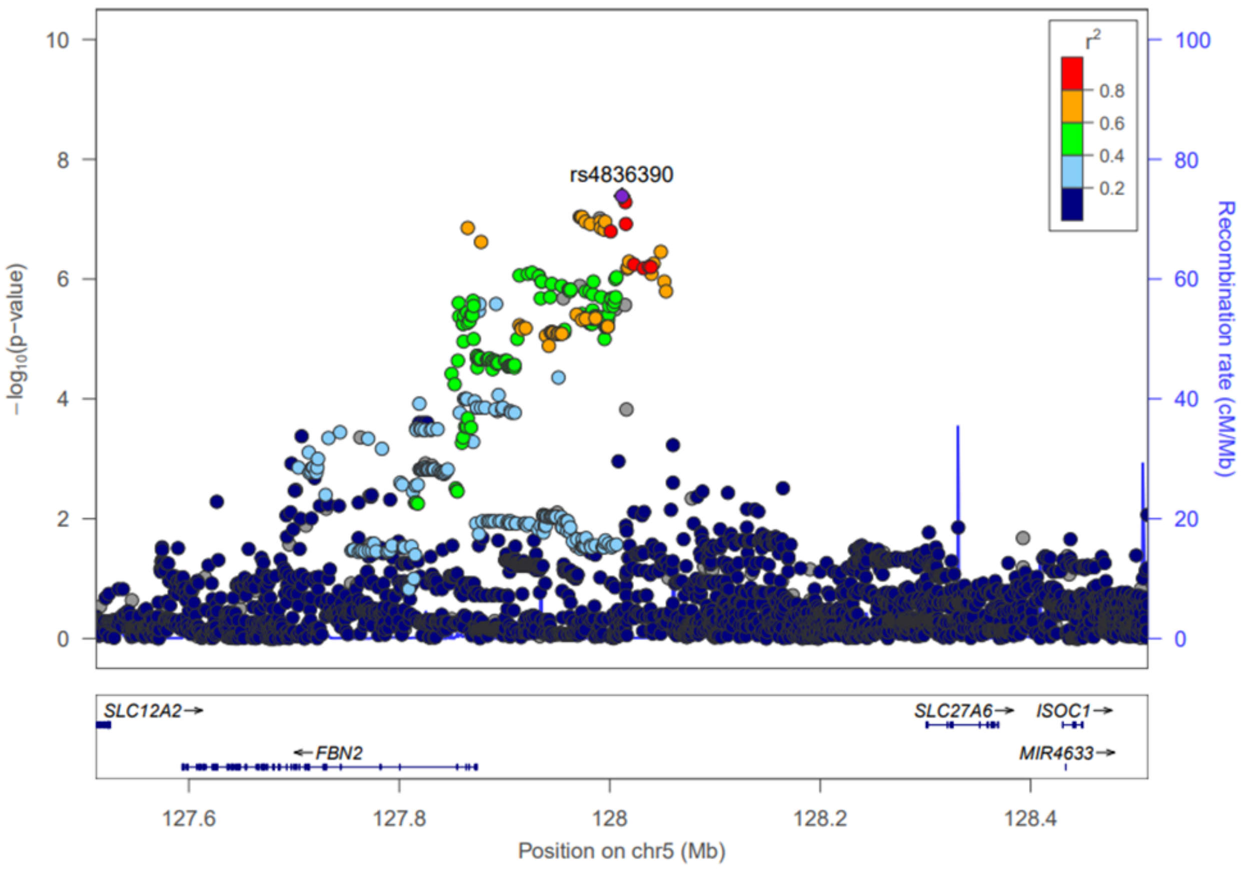

Supplementary Figure 5.38 LocusZoom plot for rs55676951 from GWAS for PC9

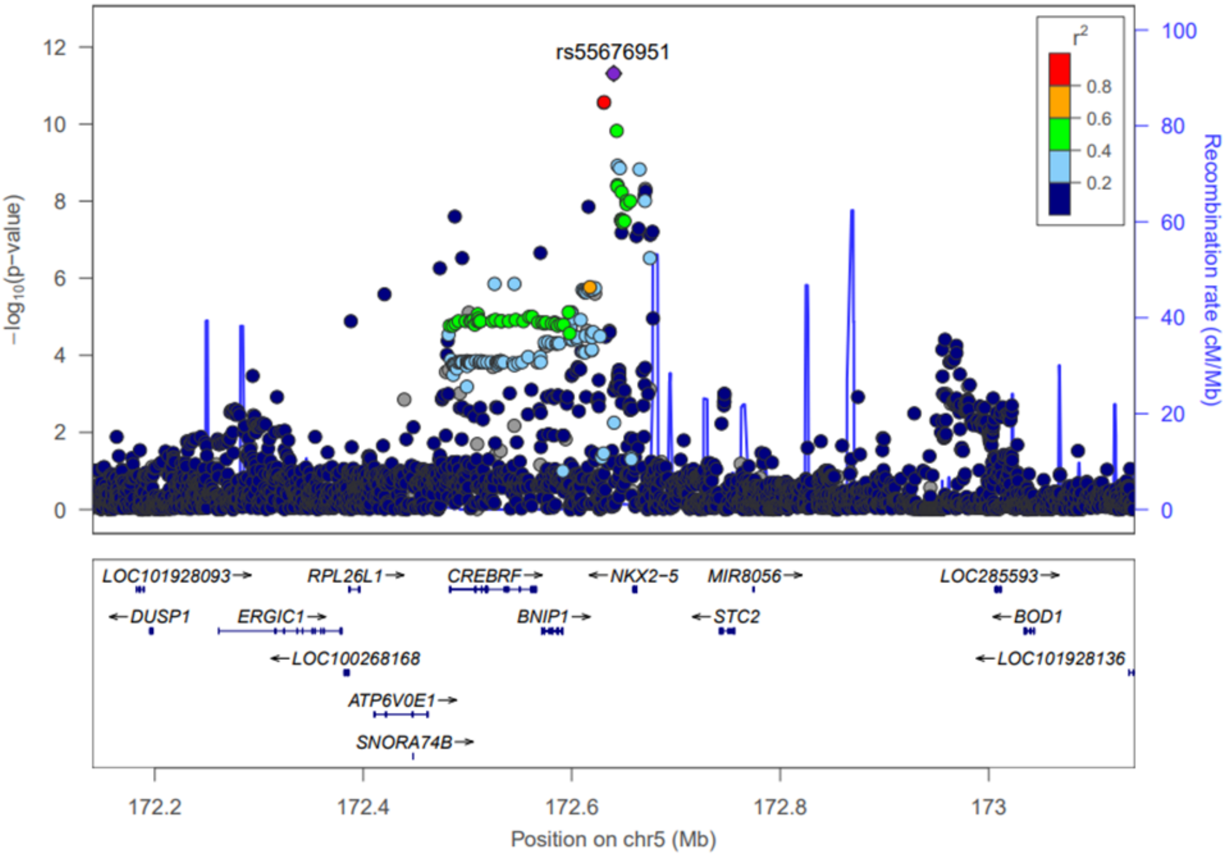

Supplementary Figure 5.39 LocusZoom plot for rs56403768 from GWAS for PC9

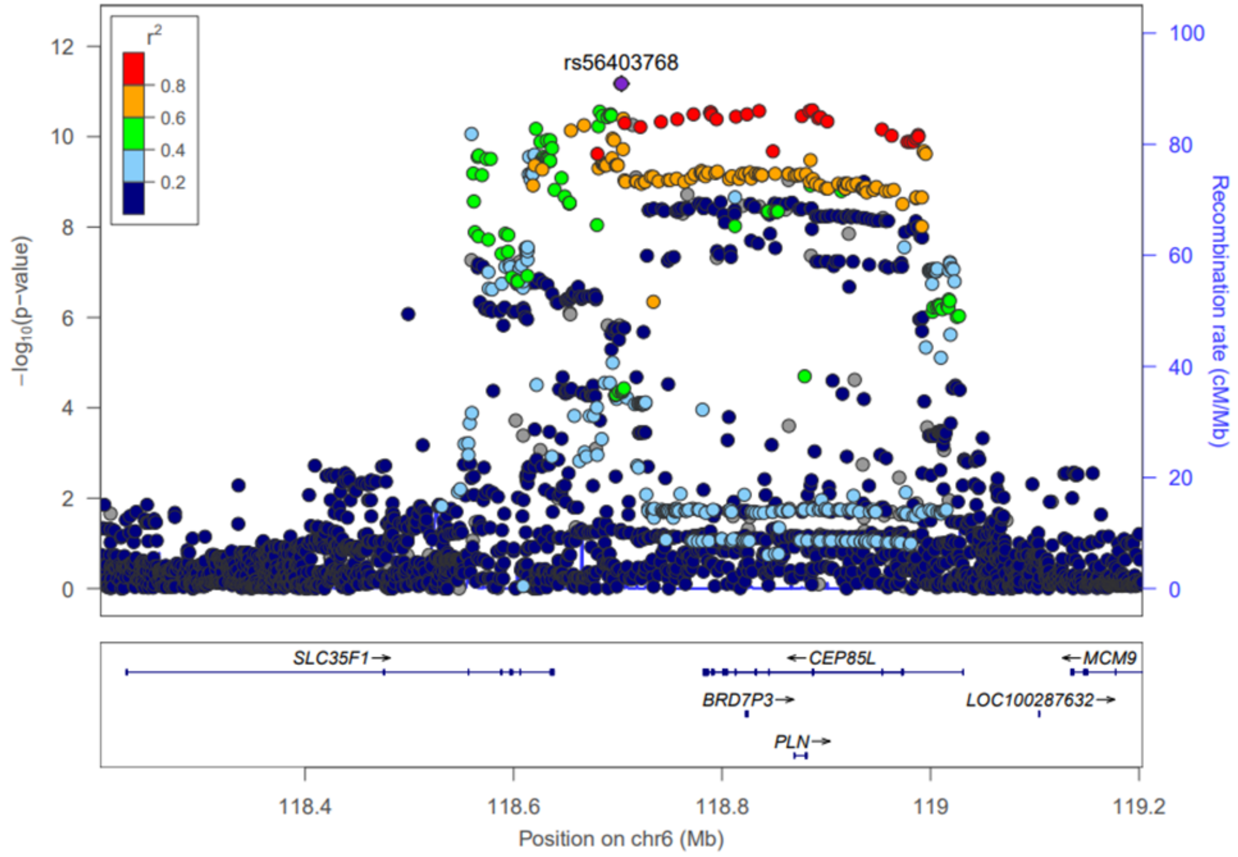

Supplementary Figure 5.40 LocusZoom plot for rs10918594 from GWAS for PC10

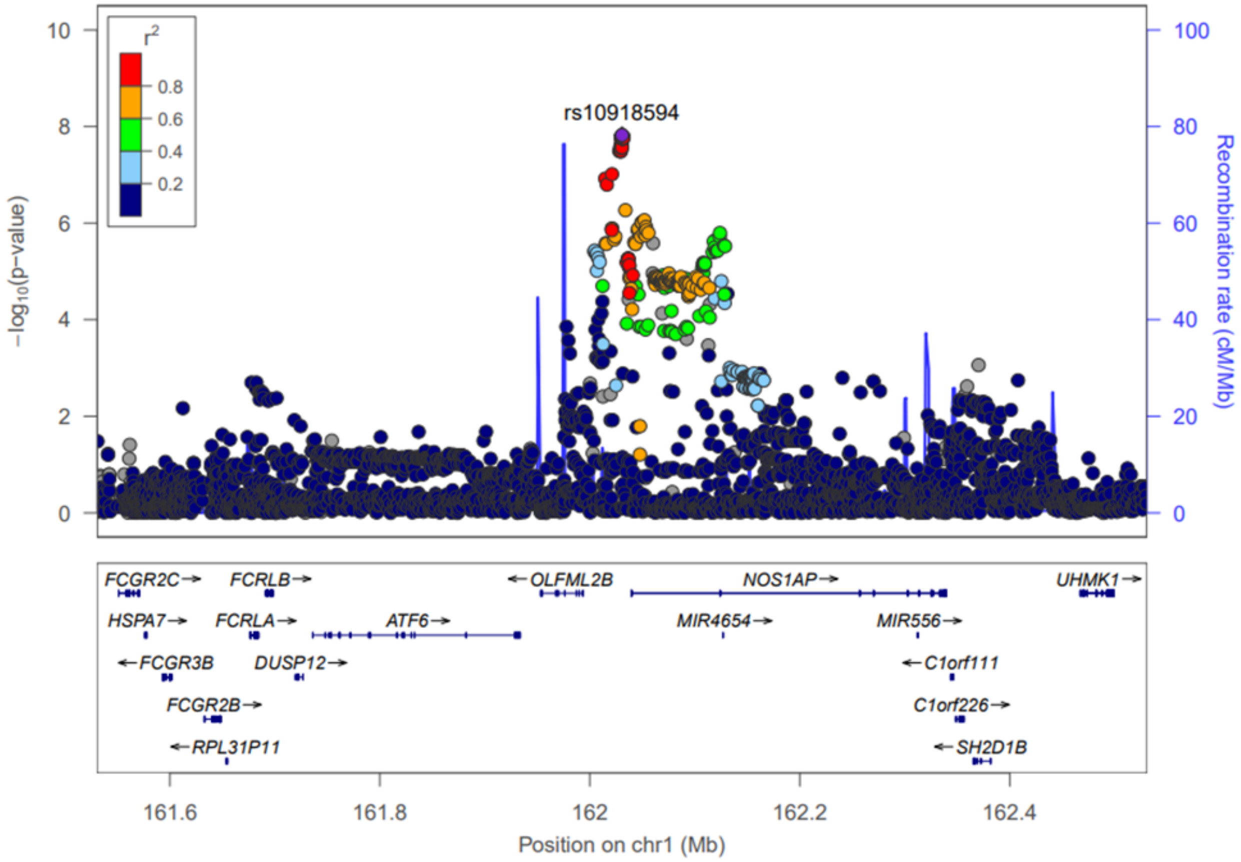

GWAS, genome wide association study; Chr, chromosome; each point symbolises a single nucleotide variant (SNV), colour coded (legend) based on linkage disequilibrium (r2) with the lead (purple) where available. Where no LD information is available for an SNV, the plot is grayscale. Recombination rate marked by blue peaks along the X-axis, measures using the right Y-axis. Annotated genes are presented below the plot, along with their base position in the chromosome labelled.
