# Supplementary material for: Genetic basis of right and left ventricular heart shape
Source: Nat Commun. 2024 Nov 14;15:9437. doi: 10.1038/s41467-024-53594-7 (PMC11564811; doi:10.1038/s41467-024-53594-7)
Supplement: Supplementary file 3 — Description of Additional Supplementary Files [file 41467_2024_53594_MOESM3_ESM.pdf]

Description of Additional Supplementary Files

Supplementary Movies

Supplementary Movie 1

PC1 shape variation from the anterior view

A plot of PC1 cycling between +2 and -2 standard deviations from the mean shape from the anterior view. Left ventricle is shaded green, right ventricle is shaded blue, and the myocardium is shaded pink.

Supplementary Movie 2

PC1 shape variation from the basal view

A plot of PC1 cycling between +2 and -2 standard deviations from the mean shape from the basal view. Left ventricle is shaded green, right ventricle is shaded blue, and the myocardium is shaded pink.

Supplementary Movie 3

PC1 shape variation from the posterior view

A plot of PC1 cycling between +2 and -2 standard deviations from the mean shape from the posterior view. Left ventricle is shaded green, right ventricle is shaded blue, and the myocardium is shaded pink.

Supplementary Movie 4

PC2 shape variation from the anterior view

A plot of PC2 cycling between +2 and -2 standard deviations from the mean shape from the anterior view. Left ventricle is shaded green, right ventricle is shaded blue, and the myocardium is shaded pink.

Supplementary Movie 5

PC2 shape variation from the basal view

A plot of PC2 cycling between +2 and -2 standard deviations from the mean shape from the basal view. Left ventricle is shaded green, right ventricle is shaded blue, and the myocardium is shaded pink.

Supplementary Movie 6

PC2 shape variation from the posterior view

A plot of PC2 cycling between +2 and -2 standard deviations from the mean shape from the posterior view. Left ventricle is shaded green, right ventricle is shaded blue, and the myocardium is shaded pink.

Supplementary Movie 7

PC3 shape variation from the anterior view

A plot of PC3 cycling between +2 and -2 standard deviations from the mean shape from the anterior view. Left ventricle is shaded green, right ventricle is shaded blue, and the myocardium is shaded pink.

Supplementary Movie 8

PC3 shape variation from the basal view

A plot of PC3 cycling between +2 and -2 standard deviations from the mean shape from the basal view. Left ventricle is shaded green, right ventricle is shaded blue, and the myocardium is shaded pink.

Supplementary Movie 9

PC3 shape variation from the posterior view

A plot of PC3 cycling between +2 and -2 standard deviations from the mean shape from the posterior view. Left ventricle is shaded green, right ventricle is shaded blue, and the myocardium is shaded pink.

Supplementary Movie 10

PC4 shape variation from the anterior view

A plot of PC4 cycling between +2 and -2 standard deviations from the mean shape from the anterior view. Left ventricle is shaded green, right ventricle is shaded blue, and the myocardium is shaded pink.

Supplementary Movie 11

PC4 shape variation from the basal view

A plot of PC4 cycling between +2 and -2 standard deviations from the mean shape from the basal view. Left ventricle is shaded green, right ventricle is shaded blue, and the myocardium is shaded pink.

Supplementary Movie 12

PC4 shape variation from the posterior view

A plot of PC4 cycling between +2 and -2 standard deviations from the mean shape from the posterior view. Left ventricle is shaded green, right ventricle is shaded blue, and the myocardium is shaded pink.

Supplementary Movie 13

PC5 shape variation from the anterior view

A plot of PC5 cycling between +2 and -2 standard deviations from the mean shape from the anterior view. Left ventricle is shaded green, right ventricle is shaded blue, and the myocardium is shaded pink.

Supplementary Movie 14

PC5 shape variation from the basal view

A plot of PC5 cycling between +2 and -2 standard deviations from the mean shape from the basal view. Left ventricle is shaded green, right ventricle is shaded blue, and the myocardium is shaded pink.

Supplementary Movie 15

PC5 shape variation from the posterior view

A plot of PC5 cycling between +2 and -2 standard deviations from the mean shape from the posterior view. Left ventricle is shaded green, right ventricle is shaded blue, and the myocardium is shaded pink.

Supplementary Movie 16

PC6 shape variation from the anterior view

A plot of PC6 cycling between +2 and -2 standard deviations from the mean shape from the anterior view. Left ventricle is shaded green, right ventricle is shaded blue, and the myocardium is shaded pink.

Supplementary Movie 17

PC6 shape variation from the basal view

A plot of PC6 cycling between +2 and -2 standard deviations from the mean shape from the basal view. Left ventricle is shaded green, right ventricle is shaded blue, and the myocardium is shaded pink.

Supplementary Movie 18

PC6 shape variation from the posterior view

A plot of PC6 cycling between +2 and -2 standard deviations from the mean shape from the posterior view. Left ventricle is shaded green, right ventricle is shaded blue, and the myocardium is shaded pink.

Supplementary Movie 19

PC7 shape variation from the anterior view

A plot of PC7 cycling between +2 and -2 standard deviations from the mean shape from the anterior view. Left ventricle is shaded green, right ventricle is shaded blue, and the myocardium is shaded pink.

Supplementary Movie 20

PC7 shape variation from the basal view

A plot of PC7 cycling between +2 and -2 standard deviations from the mean shape from the basal view. Left ventricle is shaded green, right ventricle is shaded blue, and the myocardium is shaded pink.

Supplementary Movie 21

PC7 shape variation from the posterior view

A plot of PC7 cycling between +2 and -2 standard deviations from the mean shape from the posterior view. Left ventricle is shaded green, right ventricle is shaded blue, and the myocardium is shaded pink.

Supplementary Movie 22

PC8 shape variation from the anterior view

A plot of PC8 cycling between +2 and -2 standard deviations from the mean shape from the anterior view. Left ventricle is shaded green, right ventricle is shaded blue, and the myocardium is shaded pink.

Supplementary Movie 23

PC8 shape variation from the basal view

A plot of PC8 cycling between +2 and -2 standard deviations from the mean shape from the basal view. Left ventricle is shaded green, right ventricle is shaded blue, and the myocardium is shaded pink.

Supplementary Movie 24

PC8 shape variation from the posterior view

A plot of PC8 cycling between +2 and -2 standard deviations from the mean shape from the posterior view. Left ventricle is shaded green, right ventricle is shaded blue, and the myocardium is shaded pink.

Supplementary Movie 25

PC9 shape variation from the anterior view

A plot of PC9 cycling between +2 and -2 standard deviations from the mean shape from the anterior view. Left ventricle is shaded green, right ventricle is shaded blue, and the myocardium is shaded pink.

Supplementary Movie 26

PC9 shape variation from the basal view

A plot of PC9 cycling between +2 and -2 standard deviations from the mean shape from the basal view. Left ventricle is shaded green, right ventricle is shaded blue, and the myocardium is shaded pink.

Supplementary Movie 27

PC9 shape variation from the posterior view

A plot of PC9 cycling between +2 and -2 standard deviations from the mean shape from the posterior view. Left ventricle is shaded green, right ventricle is shaded blue, and the myocardium is shaded pink.

Supplementary Movie 28

PC10 shape variation from the anterior view

A plot of PC10 cycling between +2 and -2 standard deviations from the mean shape from the anterior view. Left ventricle is shaded green, right ventricle is shaded blue, and the myocardium is shaded pink.

Supplementary Movie 29

PC10 shape variation from the basal view

A plot of PC10 cycling between +2 and -2 standard deviations from the mean shape from the basal view. Left ventricle is shaded green, right ventricle is shaded blue, and the myocardium is shaded pink.

Supplementary Movie 30

PC10 shape variation from the posterior view

A plot of PC10 cycling between +2 and -2 standard deviations from the mean shape from the posterior view. Left ventricle is shaded green, right ventricle is shaded blue, and the myocardium is shaded pink.

Supplementary Movie 31

PC11 shape variation from the anterior view

A plot of PC11 cycling between +2 and -2 standard deviations from the mean shape from the anterior view. Left ventricle is shaded green, right ventricle is shaded blue, and the myocardium is shaded pink.

Supplementary Movie 32

PC11 shape variation from the basal view

A plot of PC11 cycling between +2 and -2 standard deviations from the mean shape from the basal view. Left ventricle is shaded green, right ventricle is shaded blue, and the myocardium is shaded pink.

Supplementary Movie 33

PC11 shape variation from the posterior view

A plot of PC11 cycling between +2 and -2 standard deviations from the mean shape from the posterior view. Left ventricle is shaded green, right ventricle is shaded blue, and the myocardium is shaded pink.

Supplementary Data Files

Supplementary Data 1
Cohort characteristics of the UK Biobank from the imaging cohort

Supplementary Data 2
Correlation test results for shape PCs and cardiovascular structure and functional measures from CMR and cardiovascular risk factors ($p < 0.05$)

Supplementary Data 3
Association results from PC and PC polygenic risk scores with atrial fibrillation

Supplementary Data 4
Association results from PC and PC polygenic risk scores with dilated cardiomyopathy

Supplementary Data 5
Association results from PC and PC polygenic risk scores with diabetes mellitus

Supplementary Data 6
Association results from PC and PC polygenic risk scores with hypertrophic cardiomyopathy

Supplementary Data 7
Association results from PC and PC polygenic risk scores with heart failure

Supplementary Data 8
Association results from PC and PC polygenic risk scores with myocardial infarction

Supplementary Data 9
Association results from PC and PC polygenic risk scores with atrioventricular block

Supplementary Data 10
Lookup of lead SNV's and proxies in GWAS summary statistics

Supplementary Data 11
Significant genes identified by MAGMA ($p < 2.6x10^{-6}$)

Supplementary Data 12
VEP variant level annotations of the moderate/high impact variants from the shape GWAS

Supplementary Data 13
eQTL and colocalisation results for lead and high LD variants from shape GWAS

Supplementary Data 14
Transcriptome-wide association study results from S-PREDIXCAN ($p < 3.1x10^{-6}$)

Supplementary Data 15
Long-range chromatin interaction analysis of the shape loci using FUMA

Supplementary Data 16
Long-range chromatin interaction analysis of the shape loci using Jung dataset

Supplementary Data 17
GARFIELD hypersensitivity site tissue enrichments

Supplementary Data 18
A table of the candidate genes and their supporting evidence

Supplementary Data 19
FUMA Gene2Func results for individual PCs (FDR rate $p < 0.05$)

Supplementary Data 20
Results from FUMA Gene2Func tool with significance FDR $p < 0.05$ for all GWS SNV's

Supplementary Data 21
GWAS catalog results for significant SNV's and their proxies ($r^2 > 0.8$)

Supplementary Data 22
Phenoscanner results for significant SNV's and their proxies ($r^2 > 0.8$)

Supplementary Data 23
Lookups of lead and secondary signals and loci in existing data (as of September 2022)

Supplementary Data 24
Results of the PheWAS analysis using the lead and conditionally independent SNV's

Supplementary Data 25
Effect allele frequency across ancestries from each SNV included in the PRS

Supplementary Data 26
Percent variance explained by PRS

Supplementary Data 27
ICD classifications used in study outcome definition

Abbreviations:
