# Supplementary material for: Genetic basis of right and left ventricular heart shape
Source: Nat Commun. 2024 Nov 14;15:9437. doi: 10.1038/s41467-024-53594-7 (PMC11564811; doi:10.1038/s41467-024-53594-7)
Supplement: Supplementary file 38 — Reporting Summary [file 41467_2024_53594_MOESM38_ESM.pdf]

nature portfolio

Corresponding author(s): Young and Munroe, NCOMMS-23-10979-T

Last updated by author(s): 9/6/2024

Reporting Summary

Nature Portfolio wishes to improve the reproducibility of the work that we publish. This form provides structure for consistency and transparency in reporting. For further information on Nature Portfolio policies, see our Editorial Policies and the Editorial Policy Checklist.

Statistics

For all statistical analyses, confirm that the following items are present in the figure legend, table legend, main text, or Methods section.

n/a	Confirmed	
☐	☒	The exact sample size (*n*) for each experimental group/condition, given as a discrete number and unit of measurement
☐	☒	A statement on whether measurements were taken from distinct samples or whether the same sample was measured repeatedly
☐	☒	The statistical test(s) used AND whether they are one- or two-sided<br>*Only common tests should be described solely by name; describe more complex techniques in the Methods section.*
☐	☒	A description of all covariates tested
☐	☒	A description of any assumptions or corrections, such as tests of normality and adjustment for multiple comparisons
☐	☒	A full description of the statistical parameters including central tendency (e.g. means) or other basic estimates (e.g. regression coefficient) AND variation (e.g. standard deviation) or associated estimates of uncertainty (e.g. confidence intervals)
☒	☐	For null hypothesis testing, the test statistic (e.g. *F*, *t*, *r*) with confidence intervals, effect sizes, degrees of freedom and *P* value noted<br>*Give P values as exact values whenever suitable.*
☒	☐	For Bayesian analysis, information on the choice of priors and Markov chain Monte Carlo settings
☐	☒	For hierarchical and complex designs, identification of the appropriate level for tests and full reporting of outcomes
☐	☒	Estimates of effect sizes (e.g. Cohen's *d*, Pearson's *r*), indicating how they were calculated

Our web collection on statistics for biologists contains articles on many of the points above.

Software and code

Policy information about availability of computer code

Data collection	Circle cvi42 version 5.11

Data analysis	R - v4.2.2<br>BOLT-REML - v2.4.1<br>BOLT-LMM - v2.4.1<br>PLINK - v1.9<br>LDSC - v1.0.1<br>GCTA - v1.94.1<br>MAGMA - v1.06<br>VEP - release 105.0<br>SIFT - v5.2.2<br>PolyPhen-2 - v2.2.2 release 405c<br>S-PREDIXCAN - v0.6.5<br>GARFIELD - v2<br>FUMA - v1.4.0<br>PheWAS - v0.99.5-5<br>PRSICE2 - v2.3.5<br>COLOC - v5.1.0.1<br>locuszoom .js<br>TwoSampleMR R package (version 0.6.6)

For manuscripts utilizing custom algorithms or software that are central to the research but not yet described in published literature, software must be made available to editors and reviewers. We strongly encourage code deposition in a community repository (e.g. GitHub). See the Nature Portfolio guidelines for submitting code & software for further information.

Data

Policy information about availability of data

All manuscripts must include a data availability statement. This statement should provide the following information, where applicable:

- Accession codes, unique identifiers, or web links for publicly available datasets
- A description of any restrictions on data availability
- For clinical datasets or third party data, please ensure that the statement adheres to our policy

The main summary statistics will be made available on the GWAS catalog on publication (https://www.ebi.ac.uk/gwas/).

Data relating to UK Biobank will be returned to the study. The UK Biobank will make these data available to all bona fide researchers for all types of health-related research that is in the public interest, without preferential or exclusive access for any person. All researchers will be subject to the same application process and approval criteria as specified by the UK Biobank. Please see the UK Biobank's website for the detailed access procedure (http:/www.ukbiobank.ac.uk/register-apply/).

Research involving human participants, their data, or biological material

Policy information about studies with human participants or human data. See also policy information about sex, gender (identity/presentation), and sexual orientation and race, ethnicity and racism.

Reporting on sex and gender	The authors report on sex only and the information on sex is made available through the UK Biobank, and is acquired from central registry at recruitment, but in some cases updated by the participant. Hence the field used in this study may contain a mixture of the sex the NHS had recorded for the participant and self-reported sex. Sex is used as a covariate in statistical analyses only.
Reporting on race, ethnicity, or other socially relevant groupings	In this study there is focus on an individuals ethnic ancestry at several points of the study, referred to as ancestry. We restrict to European ethnic ancestry in some analyses, as this is the predominant ancestry available in the UK Biobank and there are not substantial numbers of other ancestries for comparable study power. Ancestry in this study is defined as the agreement between the self reported ethnicity a participant indicates at assessment centre visit as part of the touchscreen questionnaire, and the genetically inferred ancestry which is derived from a k-means clustering method which uses genetic principal components to cluster participants into 4 main distinct ethnic groups: European, African, East Asian (Chinese) and South Asian.
Population characteristics	These analyses were performed on individuals recruited to the UK Biobank - a prospective population study. For validation of one of the PRS we studied a clinical cohort with hypertrophic cardiomyopathy (HCM), full details are provided in the methods.
Recruitment	Information on recruitment and selection criteria are provided in the methods for both UK Biobank and the HCM cohort.
Ethics oversight	This study complies with the Declaration of Helsinki; the work was covered by the ethical approval for UK Biobank studies from the NHS National Research Ethics Service on 17th June 2011 (Ref 11/NW/0382) and extended on 18 June 2021 (Ref 21/NW/0157) with written informed consent obtained from all participants. HCM cohort study approved by the regional ethics committees (London: 15/LO/0549; Coruna: 2021/182).

Note that full information on the approval of the study protocol must also be provided in the manuscript.

Field-specific reporting

Please select the one below that is the best fit for your research. If you are not sure, read the appropriate sections before making your selection.

- [x] Life sciences
- [ ] Behavioural & social sciences
- [ ] Ecological, evolutionary & environmental sciences

For a reference copy of the document with all sections, see nature.com/documents/nr-reporting-summary-flat.pdf

Life sciences study design

All studies must disclose on these points even when the disclosure is negative.

Sample size	This study aimed to discover the genetic basis of cardiac shape features, the largest sample size in UK Biobank was used, no power calculations were performed. For the validation of the PRS for PC5 and HCM, we used an external cohort that we derived age and sex matched case controls from the UK Biobank. These were drawn from a random sampling without replacement of the UK Biobank for each HCM patient in the external cohort matched to their age by decade and sex. Decade was chosen as the sample has several younger patients which we were unable to derive case controls without replacement when looking for an exact age match. The study aimed for a case control of 20:1, with 2313 HCM cases, however after exclusions based on availability of whole exome sequencing data and exclusion of individuals present in the imaging study (detailed in the methods) the resulting control number was 40825, at a ratio of 17.7:1
Data exclusions	In the construction of the atlas we exclude UK Biobank CMR imaging studies with incomplete data, with image modalities mis-classified by the contouring software and cases in which the software was unable to provide a complete set of contour points.<br>We implement a stringent quality control method to the models created for the atlas (described in supplementary methods) to identify good quality models, and combine these with quality control methods for the genetics cohort to define our final study cohort.<br>For the GWAS on an individual level we exclude non-white European individuals defined by agreement between self reported and genetically inferred ancestry definition, participants with previous major adverse cardiovascular events (detailed in supplementary methods) or with a pathologically low ejection fraction (<40%), or with missing covariate data.<br>On a variant level, we exclude variants with a minor allele frequency < 1%, a Hardy-Weinberg exact test > 1x10-6 or a missing rate >1.5%. Further information is available in the methods section. Participants included in the GWAS were excluded from subsequent calculations of the PRS score associations.
Replication	We performed a replication study for the PC5 PRS and HCM status in an external dataset, full details are provided in methods and results.
Randomization	Randomisation was not necessary for these analyses.
Blinding	Blinding was not relevant to this study.

Reporting for specific materials, systems and methods

We require information from authors about some types of materials, experimental systems and methods used in many studies. Here, indicate whether each material, system or method listed is relevant to your study. If you are not sure if a list item applies to your research, read the appropriate section before selecting a response.

Materials & experimental systems

n/a	Involved in the study
☒	☐ Antibodies
☒	☐ Eukaryotic cell lines
☒	☐ Palaeontology and archaeology
☒	☐ Animals and other organisms
☒	☐ Clinical data
☒	☐ Dual use research of concern
☒	☐ Plants

Methods

n/a	Involved in the study
☒	☐ ChIP-seq
☒	☐ Flow cytometry
☒	☐ MRI-based neuroimaging
